# Glutamic acid decarboxylase 67 haplodeficiency in mice: consequences of postweaning social isolation on behavior and changes in brain neurochemical systems

**DOI:** 10.1007/s00429-020-02087-6

**Published:** 2020-06-08

**Authors:** Sven Nullmeier, Christoph Elmers, Wolfgang D’Hanis, Kiran Veer Kaur Sandhu, Oliver Stork, Yuchio Yanagawa, Patricia Panther, Herbert Schwegler

**Affiliations:** 1 Institute of Molecular and Cellular Anatomy, Ulm University, Albert-Einstein-Allee 11, 89081 Ulm, Germany; 2 Institute of Anatomy, Otto-Von-Guericke University Magdeburg, Leipziger Str. 44, 39120 Magdeburg, Germany; 3 Department of Genetics and Molecular Neurobiology, Institute of Biology, Otto-Von-Guericke University Magdeburg, Leipziger Str. 44, 39120 Magdeburg, Germany; 4 Department of Genetic and Behavioral Neuroscience, Gunma University Graduate School of Medicine, 3-39-22 Showa-machi, Maebashi, Gunma, 371-8511 Japan; 5 Department of Neurosurgery, Ulm University Medical Center, Albert-Einstein-Allee 23, 89081 Ulm, Germany; 6 Center for Behavioral Brain Sciences, Universitätsplatz 2, 39106 Magdeburg, Germany

**Keywords:** GAD67, Hippocampus, Schizophrenia, Social interaction, Social isolation, Dopamine

## Abstract

Reductions of glutamate acid decarboxylase (GAD67) and subsequent GABA levels have been consistently observed in neuropsychiatric disorders like schizophrenia and depression, but it has remained unclear how GABAergic dysfunction contributes to different symptoms of the diseases. To address this issue, we investigated male mice haplodeficient for GAD67 (*GAD67*^+*/GFP*^ mice), which showed a reduced social interaction, social dominance and increased immobility in the forced swim test. No differences were found in rotarod performance and sensorimotor gating. We also addressed potential effects of social deprivation, which is known, during early life, to affect GABAergic function and induces behavioral abnormalities similar to the symptoms found in psychiatric disorders. Indeed, social isolation of *GAD67*^+*/GFP*^ mice provoked increased rearing activity in the social interaction test and hyperlocomotion on elevated plus maze. Since GABA closely interacts with the dopaminergic, serotonergic and cholinergic neurotransmitter systems, we investigated *GAD67*^+*/GFP*^ and *GAD67*^+*/*+^ mice for morphological markers of the latter systems and found increased tyrosine hydroxylase (TH)-IR fiber densities in CA1 of dorsal hippocampus. By contrast, no differences in numbers and densities of TH-positive neurons of the midbrain dopamine regions, serotonin (5-HT) neurons of the raphe nuclei, or choline acetyltransferase (ChAT)-expressing neurons of basal forebrain and their respective terminal fields were observed. Our results indicate that GAD67 haplodeficiency impairs sociability and increases vulnerability to social stress, provokes depressive-like behavior and alters the catecholaminergic innervation in brain areas associated with schizophrenia. *GAD67*^+*/GFP*^ mice may provide a useful model for studying the impact of GABAergic dysfunction as related to neuropsychiatric disorders.

**Electronic supplementary material:**

The online version of this article (10.1007/s00429-020-02087-6) contains supplementary material, which is available to authorized users.

## Introduction

A corticolimbic GABAergic dysfunction has been implicated in the pathophysiology of neuropsychiatric disorders like schizophrenia and major depressive disorder (MDD) (Benes and Berretta 2001; Fatemi et al. 2005). The most consistent findings in post-mortem brain studies of schizophrenia patients are reduced numbers of parvalbumin (PARV)-containing GABAergic interneurons and a decreased expression of the 67-kDa isoform of the GABA-synthesizing enzyme glutamate acid decarboxylase (GAD67) in cerebral cortex and hippocampus (Akbarian et al. 1995; Guidotti et al. 2000; Hashimoto et al. 2003; Lewis et al. 2005). In contrast to GAD67, only inconsistent findings in schizophrenia are reported for the 65-kDa (GAD65) isoform (Guidotti et al. 2000; Glausier et al. 2015; de Jonge et al. 2017). Besides GABAergic dysfunction, alterations of other neurotransmitter pathways in different brain areas have been reported (Brisch et al. 2014). Regarding the dopaminergic (DAergic) system, a subcortical mesolimbic hyperactivity and prefrontal mesocortical hypoactivity were postulated (Meltzer and Stahl 1976; Howes and Kapur 2009). Also, alterations of the serotonergic (5-HT) system of the raphe nuclei and dysfunctions of the cholinergic system in the basal forebrain were found to be involved in schizophrenia (Raedler et al. 2007; Geyer and Vollenweider 2008). Schizophrenia is further characterized by heterogeneous clinical symptoms including social withdrawal, avolition, hyperactivity, depressive symptoms, deficits in sensorimotor gating and increased risk for aggression (Andreasen et al. 1990; Braff et al. 2001). However, due to the etiological heterogeneity of schizophrenia the underlying molecular and cellular pathomechanisms remain largely unknown. It is assumed that schizophrenia develops through complex interactions between multiple genes and environmental factors (Lewis and Levitt 2002). In accordance with the “two-hit” hypothesis (Bayer et al. 1999), the combination of a genetically predisposition (the first hit) and an early life adverse event (the second hit) profoundly affects brain development and subsequent adult behavior, which may contribute to the occurrence of psychiatric disorders (Fone and Porkess 2008; Pietropaolo et al. 2008). Previous studies showed that post-weaning social isolation in combination with a pharmacological downregulation of the GABAergic function could act as a potential second hit (Lim et al. 2012; Gaskin et al. 2016). However, the separate role of the commonly observed GAD67 deficit as a vulnerability factor and its direct behavioral effects remain unclear. Mouse mutants deficient for GAD67 and GAD65 provide an opportunity to address specific roles of the GABAergic system in brain development and function. Since, there is little evidence for a role of GAD65 similar to that of GAD67 in human schizophrenia and null mutation of the GAD65 gene in mice develop a phenotype of increased anxiety and pathological fear memory reminiscent of posttraumatic stress disorder (Müller et al. 2015), we focused on GAD67 deficiency in mice. Homozygous *GAD67* mutants die shortly after birth due to a severe cleft palate (Asada et al. 1997). In contrast, *GAD67*^+*/GFP*^ mice, carrying a non-functional allele of the GAD67 gene due to a knock-in for the green fluorescence protein (GFP), are viable and show normal gross brain morphology (Tamamaki et al. 2003). These mice have been widely used for the identification of GABAergic neurons (Marowsky et al. 2005; Brown et al. 2008). In addition, *GAD67*^+*/GFP*^ mice display 36% reduction of GAD67 expression and 16% reduction of GABA levels in the brain of young adult (see also supplementary information and Fig. S1), whereas the level of GAD65 remains unchanged (Tamamaki et al. 2003; Wang et al. 2009). *GAD67*^+*/GFP*^ mice are also reported to show an increased vulnerability to maternal and fetal stress (Uchida et al. 2011) in association with a region specific loss of parvalbumin (PARV)-positive GABAergic neurons, similar to that observed in psychiatric patients (Uchida et al. 2014). Therefore, *GAD67*^+*/GFP*^ knock-in mice are appropriate tools to investigate the implications of GABAergic dysfunction as found in neuropsychiatric disorders. In the present study, behavioral and morphological consequences of GAD67 haplodeficiency were investigated that are potentially relevant for schizophrenia pathogenesis. Our data reveal that GAD67 haplodeficiency alone results in impaired social interaction and increases depression-like behavior. GAD67 haplodeficiency in combination with postweaning social isolation additionally provoked an increase of locomotor behavior. Further, deficiency of GAD67-mediated GABA synthesis results in an increased (TH)-positive fiber density in the hippocampus, suggesting an alteration of the catecholaminergic, presumably dopaminergic, system as a vulnerability factor downstream of the GABAergic hypofunction.

## Materials and methods

### Animals

Thirteen-to-eighteen-week-old male heterozygous *GAD67–GFP* knock-in *C57BL/6* mice (*GAD67*^+*/GFP*^ mice) in which GFP is inserted into the GAD67 gene (Tamamaki et al. 2003) and their wild-type control siblings (*GAD67*^+*/*+^) were examined in this study. Genotypes were determined with allele-specific PCR at the time of weaning as described previously (Janitzky et al. 2011). After weaning (postnatal day 21) mice from each genotype were either left in littermate groups of 2–3 or assigned to social isolation by individually housing (Makrolon cages measuring l/w/h: 27 × 21 × 14 cm, environmental enrichment with nesting material) until the beginning of the experiments. All animals were kept in a temperature, humidity, and light-controlled environment (22–25 °C, 55%, 12 h light–dark cycle, with light on at 06:00 a.m.) with free access to food and tap water. Isolated animals had only visual, auditory and olfactory contact with other isolation reared and group-housed mice. Behavioral experiments were performed during the active phase and body weight was measured before each experiment. All experiments were carried out in accordance with the European Communities Council Directive (2010/63/EU) and approved by the local authorities of Sachsen-Anhalt/Germany (AZ: 42502-2-1134 UniMD).

### Social interaction test in the open field

The social interaction test was performed using pairs of unfamiliar strain mates (isolated *GAD67*^+*/GFP*^ (*n* = 9) and *GAD67*^+*/*+^ (*n* = 8) and group-housed *GAD67*^+*/GFP*^ (*n* = 9) and *GAD67*^+*/*+^ (*n* = 10)). Mice were tested in a light gray upwardly open plastic arena (l/w/h: 85 × 85 × 30 cm) with a homogenous, shadow free and bright neon-illumination (300 lx). Each experiment was recorded with a ceiling mounted camera (Panasonic CCTV Camera; Mod. WVBL200/6) and analyzed using Videomot2 video-tracking software (TSE, Homburg, Germany). At the beginning of each 10-min trial, two unfamiliar mice from the same genotype and housing condition (group housed or isolated) were placed into opposite corners of the open field, recorded, and automatically analyzed by the software for various parameters (Table 1). Analysis of distance traveled was carried out as a sign of motor activity, anxiety like behavior as time spent in center region. Afterwards, the coded video sequences were manually analyzed for a second set of parameters (Table 1). Data of the individual pairs were used for statistics. With self-compounded software, using fuzzy logic, collected raw data were additionally processed to compensate weaknesses of the image recognition software (Wolf et al. 2018).

**Table 1:** Social interaction test in the open field

Analyzed parameters during the experiment	
1	Time spent in contact in percent of experimental time [%]_2_
2	Number of contacts [*n*]_2_
3	Time spent in aggressivity in percent of experimental time [%]_2_
4	Number of aggressive contacts_2_
5	Mean distance between the two mice [cm]_1_
6	Active social interaction, in percent of experimental time [%] during a movement speed higher than 3 cm/s_1_
7	Passive social interaction, in percent of experimental time [%] during a movement speed lower than 1 cm/s_1_
8	Sniffing [*n*]_2_
9	Anogenital sniffing [*n*]_2_
10	Following [*n*]_2_
11	Rearing [*n*]_2_
12	Leaning [*n*]_2_
13	Grooming [*n*]_2_
14	Distance traveled [m]_1_
15	Time spent in the center or periphery of the open field in percent of experimental time [%]_1_

Automatically (1) and manually (2) analyzed parameters in the social interaction test

### Social dominance tube test

To test social dominance a custom-made tube (transparent acrylic glass, 35 cm long and 3 cm diameter) was used. Each animal (group-housed *GAD67*^+*/GFP*^ (*n* = 10) and *GAD67*^+*/*+^ (*n* = 10) and isolated *GAD67*^+*/GFP*^ (*n* = 10) and *GAD67*^+*/*+^ (*n* = 10)) was given a pre-competition habituation period of three training trials on each of three consecutive days. Driven by food deprivation (85% of normal weight) the animals run through the tube to a food reward in a goal box (l/w/h: 15 × 9 × 9 cm). For each trial, animals were alternated in the direction that they ran through the tube. During the experiments one *GAD67*^+*/GFP*^ and one unfamiliar *GAD67*^+*/*+^mouse from same housing condition were placed at opposite ends of the tube, in a head-to-head direction and released. A mouse was declared a “winner” when its opponent backed out with all four paws of the tube. The maximal test time was set to 2 min. Each mouse was tested for a total of seven times on two consecutive days, each time against an unfamiliar opponent (70 trails). The number of wins and losses for each genotype was determined.

### Rotarod test

To assess mild neurological deficits such as impaired motor learning and coordination in rodents we used a mouse rotarod (model 47600, Ugo Basile, Comerio, Italy). One day prior the experiments mice (isolated *GAD67*^+*/GFP*^ (*n *= 12) and *GAD67*^+*/*+^ (*n* = 12) and group-housed *GAD67*^+*/GFP*^ (*n* = 12) and *GAD67*^+*/*+^ (*n* = 12)) were familiarized with the rotarod at fixed speed modes of 4 and 10 rpm for 1 min, respectively. At start of the experiments, mice underwent three rotarod trials per day on three consecutive days with accelerating speed from minimum of 4 to 40 rpm over the course of each 300 s (5 min) trial. Each mouse was given an intertrial interval of 30 min. The amount of time before the mouse fell from the rod was measured.

### Forced swim test

The Porsolt forced swim test (FST) was conducted in a modified version with only one swim session. Mice (isolated *GAD67*^+*/GFP*^ (*n* = 20) and *GAD67*^+*/*+^ (*n* = 15) and group-housed *GAD67*^+*/GFP*^ (*n* = 13) and *GAD67*^+*/*+^ (*n* = 11)) were placed individually in a transparent acrylic glass cylinder (diameter 14 cm, height 21 cm) filled to a depth of 15 cm with tap water (23 °C). Each session was investigated for latency to first occurrence of immobility, time spent immobile, swimming and climbing. Only the last 4 min of the 6 min test session were analyzed by a trained observer blind to genotype. Immobility was defined as the cessation of limb movements except minor movement necessary to keep the animals head above water. Swimming was registered when large forepaw movements displaced the body around the cylinder. Climbing was defined when vigorous movements with forepaws out of the water, usually directed against the wall of the cylinder, were observed.

### Elevated plus maze

The maze consisted of a plus-shaped gray wooden platform elevated 75 cm above the floor with a center (5 cm × 5 cm) connecting the four arms (25 cm × 5 cm). Two of the opposing arms were enclosed by 35-cm-high walls (closed arms), whereas the other two arms had thin 0.3-cm-high ledges (open arms). All arms were lit by shadow free neon-illumination (mean light intensity 100 lx). At the beginning of the test, individual mice (isolated *GAD67*^+*/GFP*^ (*n* = 18) and *GAD67*^+*/*+^ (*n* = 15) and group-housed *GAD67*^+*/GFP*^ (*n* = 15) and *GAD67*^+*/*+^ (*n* = 14)) were placed in the center of the maze and allowed to explore for 10 min. Number of entries with all paws into the arms and the center as well as time spent on different positions were analyzed using a top-mounted camera and custom-made software. After finishing each session, mice were transferred back to their home cages and the maze was thoroughly wiped clean.

### Prepulse inhibition of acoustic startle response

Isolated *GAD67*^+*/GFP*^ (*n* = 19) and *GAD67*^+*/*+^ (*n* = 16) and group-housed *GAD67*^+*/GFP*^ (*n* = 14) and *GAD67*^+*/*+^ (*n* = 20) mice served for the experiments. The startle apparatus consists of a sound-attenuating chamber (l/w/h: 50 × 40 × 45 cm, illuminated by a 5 W cold light, with a movement sensitive piezo-accelerometer platform (Startle-Messsystem, University of Tübingen, Germany) on which a wire mesh test cage (l/w/h: 8 × 5 × 5 cm) was fixed. Movement-induced voltage changes were amplified (Piezo-Amp System, University of Tübingen, Germany) and digitized by data acquisition processor board DAP1200a (Microstar, Bellevue, WA) within a computer and recorded using the software program DAPview Plus^®^ (Microstar Laboratories). Startle amplitude was calculated as the difference between peak-to-peak voltage during a time window 80 ms after stimulus onset and the 80 ms before stimulus onset. The force values *F* (*N*) were converted to acceleration values *a* = *F*/*m* (m/s^2^), which represent the startle amplitudes of the animal. Thus, the results given as acceleration values are independent from animal weight (*m*). Acoustic stimuli and steady background noise (65 dB) were performed by the signal synthesizer software SigGen-PC 1.44 (Waldmann, Tübingen, Germany), generated by a stimulus generation processor board ASPI ELF-31 (Medav, Uttenreuth, Germany), amplified (WPA-600; Sony, Tokyo, Japan) and presented by a speaker located in a distance of 21 cm from the center of test platform. Each startle session consisted of 85 trials and was started with an acclimation period of ten non-stimulus trials (NOSTIM) followed by three PULSE-ALONE (113 dB SPL) trials. These trials were not included in the analysis. During the acquisition period, four different types of stimuli (Table 2) were presented in a pseudo-random order with an intertrial interval of 20 s.

**Table 2:** Parameters of acoustic startle response (ASR) and prepulse inhibition (PPI) tests

Stimulus type	Description
1. NOSTIM	No stimulus, white noise at 65 dB sound pressure level (SPL)
2. PREPULSE	Prepulse alone, sine wave, 10 kHz, 75 dB SPL, duration 20 ms, rise and fall time 0.4 ms
3. PULSE-ALONE	Startle stimulus alone, white noise at 93, 107 or 113 dB SPL, duration 20 ms, without rise and fall time
4. PREPULSE + PULSE	Startle stimulus at 93, 107 or 113 dB SPL with preceding PREPULSE at 75 dB SPL, interstimulus interval (ISI) 100 ms

Acoustic startle stimuli presented during the prepulse inhibition experiments

### Preparation of the brains for histological analyses

Group-housed male *GAD67*^+*/GFP*^ (*n* = 6) and *GAD67*^+*/*+^ (*n* = 6) mice were deeply anesthetized with an overdose of sodium pentobarbital (180 mg/kg i.p.; Merial GmbH, Hallbergmoos, Germany) and perfused transcardially with 50 ml of 0.9% saline followed by 150 ml of a mixture of 4% paraformaldehyde and 15% saturated picric acid in 0.1 M phosphate buffer pH 7.4 (PB). After decapitation, brains were rapidly dissected from the skull and postfixed for additional 5 h in the same fixative followed by 24 h storage in 20% sucrose at 4 °C. Subsequently, the brains were shock-frozen at − 40 °C in solid carbon dioxid cooled isopentane and stored at − 80 °C. The brains were cut coronally with a cryostat into four corresponding series of 40-µm-thick sections. The series were stained for 1. Nissl substance, 2. tyrosine hydroxylase (TH), 3. serotonin (5-HT) and 4. choline acetyltransferase (ChAT).

### Immunohistochemistry

For cell counting and measurement of fiber densities, the free-floating sections were stained using immunofluorescence technique against TH, 5-HT and ChAT as described elsewhere (Nullmeier et al. 2014). The following primary antibodies were used (see also supplementary Figs. S2-4): rabbit TH polyclonal antibody (dilution: 1:1000; ab112; Abcam, Cambridge, UK), rabbit 5-HT polyclonal antibody (dilution: 1:15000; catalog number: 20080; ImmunoStar, Hudson, WI, USA) and goat ChAT polyclonal antibody (dilution: 1:100; Cat. #AB144P, Millipore, Billerica, MA, USA). Unspecific bindings of polyclonal antibodies were blocked before incubation using 10% bovine serum albumin (BSA) in phosphate buffer (0.1 M, pH 7.4) and 10% of corresponding normal serum from the host of the secondary antibody. Secondary antibodies (biotinylated rabbit anti-goat IgG (BA-5000) for ChAT and biotinylated goat anti-rabbit IgG (BA-1000) for 5-HT and TH; Vector Laboratories, Burlingame, CA, USA) were used in a dilution of 1:200. Antibodies were visualized by incubation with Cy3 conjugated avidin (dilution 1:1000; Cod. no. 003-160-083; Jackson ImmunoResearch Laboratories, Baltimore Pike, PA, USA). All sections were stained in parallel to ensure comparability and minimize experimental variability. After staining, the sections were mounted on glass slides (Super Frost Plus, ThermoScientific, Germany) and coverslipped.

### Microscopy

Cell counting, area measurements and analysis of fiber density were carried out manually on coded glass slides. For microscopy and photographs, a Zeiss AxioImager.Z2 fluorescence microscope equipped with an AxioCam MRm Rev. 3.1 camera (Zeiss, Germany) and a 5× (area measurement) or 40 × objective (analyses of cell numbers and fiber densities) were used. The software Axiovision 4.8.1^®^ (Zeiss, Germany) served for image analysis and scaled measurements.

### Cell counting and area measurement

The number of ChAT-immunoreactive (IR) neurons was analyzed in the septum from bregma 1.10 mm to bregma 0.26 mm (Paxinos and Franklin 2004). The septum was subdivided into medial septum (MS, also including the horizontal and vertical diagonal band of Broca and the lambdoid septal zone) and the lateral septum (LS, also including the intermediary and ventral parts of the lateral septal nucleus). Investigation of the number of TH-positive neurons in the midbrain was carried out from bregma − 2.46 mm to bregma − 3.52 mm. The substantia nigra (SN) and ventral tegmental area (VTA) in the right hemisphere were subdivided in substantia nigra pars compacta (SNC), substantia nigra pars reticularis (SNR), substantia nigra pars lateralis (SNL) and VTA. Numbers of 5-HT-containing neurons and area measurements of the raphe nuclei were performed from bregma − 4.04 mm to bregma − 5.02 mm. Only the dorsal nucleus raphe (DR), median (MnR) and paramedian nucleus raphe (PMnR) were investigated. For each staining, all sections containing the area of interest were investigated and only cell bodies with clear neuronal shape and well-defined nucleus were counted. Additionally, neuron diameters were estimated and used for Abercrombie correction of neuron number (Abercrombie 1946). The Nissl-stained series were used to identify anatomical landmarks (Paxinos and Franklin 2004).

### Measurement of fiber density

Measurement of ChAT, TH and 5-HT-IR fiber density was performed in dorsal hippocampus, dentate gyrus (DG) and amygdala of left hemisphere. Hippocampal CA1 and CA3 regions were divided into three layers: stratum oriens (Or), stratum radiatum (Rad) and stratum lacunosum moleculare (LMol) and dentate gyrus (DG) into two layers: stratum moleculare (Mol) and stratum multiforme (ML). Measurements of dorsal hippocampus and DG started at bregma − 1.8 mm. From five consecutive slices, one image of each layer was taken, respectively. The left amygdala was divided into four subfields: lateral amygdala (La); basolateral amygdala (BLA); central amygdala nucleus, capsular part (CeC); central amygdala nucleus, medial division (CeM). Measurements started at bregma − 0.94 mm and from three consecutive slices one image of each subfield was taken. For the analysis of the scaled microphotographs, the software ImageJ (version 1.47, https://imagej.nih.gov/ij/) was used. According to previously published methods (Panther et al. 2012), fiber densities were estimated with a calibrated grid (mesh size of 10 µm) and a square (length of one side (*L*) was 80 µm). The number of planes (*P* = 9) which correspond to the vertical and horizontal lines of the grid and the margin of the square, were represented in two sets of planes (2 × *P*). The area (*A*) was calculated by *A* = *L* × 2 × *P* = 1440 µm^2^. All intersections (*n*) of fibers with the grid were counted within the square. There is a relationship between the number of points per unit area (PA) and the specific line length per unit volume (LV): PA = 1/2 × LV. If LV = 2 × PA (µm/µm^3^) and PA = n/A (counts/µm^2^) the estimated length within a volume (fiber density) was gained.

### Statistical analysis

Behavioral tests of social interaction, rotarod, FST, EPM, ASR and PPI were analyzed by 2 × 2 two-way analyses of variance (ANOVAs) using GENOTYPE (*GAD67*^+*/GFP*^ vs *GAD67*^+*/*+^) and HOUSING (isolation vs group housed) as the between-subject factors. Additional within-subject factors (e.g. trials or PULSE-ALONE intensities) were also included according the nature of the considered variables. Post hoc analyses were performed using pairwise comparisons with Bonferroni correction. For analysis of social dominance, a binary logistic model was used. Data (wins/losses) were first analyzed for an interaction of GENOTYPE × HOUSING. If no significance was achieved, GENOTYPE and HOUSING were analyzed as main effects without interaction. For histological analysis, volume was calculated by using area and section thickness. Counted neuron numbers were quantified using Abercrombie’s method (Abercrombie 1946) and then calculated from volume (mm^3^) × density (n/mm^3^). Data were normally distributed (Shapiro–Wilk test). Each brain area was analyzed, respectively, using repeated-measures ANOVAs with LAYER or REGION (two to four levels, depending on the investigated brain area) as within-subject factors and GENOTYPE (two levels: *GAD67*^+*/GFP*^ and *GAD67*^+*/*+^) as between-subject factor. Post hoc analyses were performed using unpaired *t* tests for unequal variances (Welch’s test), and adjusted *p* values were obtained via Bonferroni–Holm method. The VTA was investigated using an unpaired *t* test, respectively. Data are presented as means ± SEM. Alpha level was set at 0.05 for all main and interaction effects. The software package SPSS (IBM SPSS Statistics for Windows, Version 21.0. Armonk, NY: IBM Corp) was used for statistical analysis.

## Results

### ***Alteration of social behavior in GAD67***^+***/GFP***^*** mice***

**Fig. 1:**
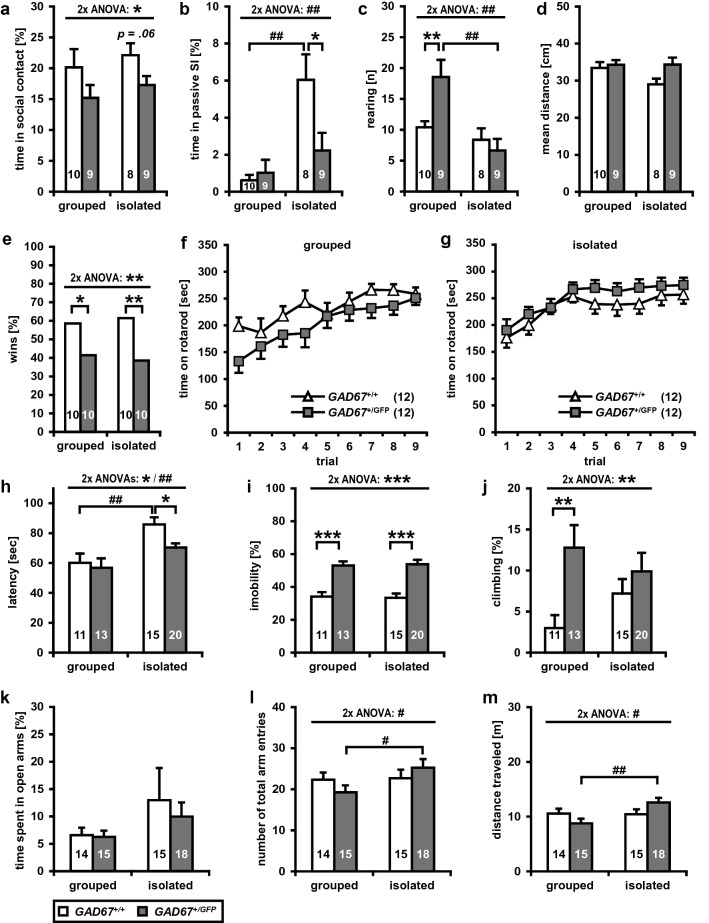
*GAD67*^+*/GFP*^ mice exhibit a subset of negative symptom-like behavioral deficits. **a**–**d** In the social interaction test **a** genotype had a significant main effect on time spent in social contact. However, only isolated *GAD67*^+*/GFP*^ mice, compared to isolated *GAD67*^+*/*+^, showed a trend towards a lower time spent in social contact. **b** Social isolation had an effect on passive social interaction. Socially isolated *GAD67*^+*/*+^ mice spent significantly more time in passive social interaction than group-housed GAD67^+/+^ mice. Isolated *GAD67*^+*/GFP*^ showed a significantly lower passive social interaction than socially isolated *GAD67*^+*/*+^ mice. **c** Social isolation had a significant effect on rearing activity. Isolated *GAD67*^+*/GFP*^ mice revealed a lower rearing activity than group-housed *GAD67*^+*/GFP*^. Group-housed *GAD67*^+*/GFP*^ showed a significantly higher rearing activity compared with group-housed *GAD67*^+*/*+^. **d** There were no differences in locomotor activity. **e** In social dominance tube test isolated and grouped *GAD67*^+*/GFP*^ lost significantly more bouts, than *GAD67*^+*/*+^ mice. **f**, **g** Genotype and housing showed no effect on rotarod performance. **h**–**j** In the forced swim test significant main effects for genotype and housing were found for latency to appearance of first immobility period. **h** Isolated *GAD67*^+*/GFP*^, compared to isolated *GAD67*^+*/*+^ mice showed a significantly reduced latency. Interestingly, social isolation increased the latency of *GAD67*^+*/*+^ mice. **i***GAD67*^+*/GFP*^ exhibit a significantly increased time spent in immobility, independent from housing condition. **j** The genotype showed an effect on climbing activity. Group-housed *GAD67*^+*/GFP*^ mice revealed a significantly higher climbing activity, compared to *GAD67*^+*/*+^. **k**–**m** In the elevated plus maze test, no differences were found for **k** time spent in open arms. **l** The number of total arm entries was dependent on the housing condition. Social isolation significantly increased the number of total arms entries, of *GAD67*^+*/GFP*^ mice. **m** Social isolation had an effect on locomotor activity on EPM. Social isolation significantly increased the distance traveled in *GAD67*^+*/GFP*^ mice. Statistics: Animal number *n* is indicated in parentheses or plot bars. Data are presented as mean ± SEM. The social interaction test, forced swim test, elevated plus maze rotarod were analyzed using two-way multivariate analyses of variance (MANOVA) or repeated measures ANOVA with GENOTYPE (two levels: *GAD67*^+*/*+^ and *GAD67*^+*/GFP*^) and HOUSING (two levels: group housed and isolated) as between-subject factors. Post hoc analyses were performed using unpaired t tests. The social dominance tube test was analyzed using a binary logistic model followed by Wald Chi-square test. *p < 0.05, **p < 0.01, ***p < 0.001 vs. *GAD67*^+*/*+^ mice. ^*#*^*p* < 0.05, ^*##*^*p* < 0.01 vs. group-housed mice

In the social interaction test (SI) pairs of male *GAD67*^+*/GFP*^ and *GAD67*^+*/*+^ from same genotype and housing condition, respectively, were analyzed for different parameters (see Table 1, Fig. 1a–d). A two-way multivariate ANOVA (between-subject factors: GENOTYPE and HOUSING) revealed significant interactions of GENOTYPE × HOUSING for passive social interaction (*F*(1, 32) = 5.94, *p* < 0.05) and rearing (*F*(1, 32) = 6.38, *p* < 0.05). GENOTYPE showed a significant difference in time spent in social contact (*F*(1, 32) = 4.74, *p* < 0.05), revealing a reduction in social contacts for *GAD67*^+*/GFP*^ mice (Fig. 1a). Only trends towards a significant main effect for passive social interaction (*F*(1, 32) = 3.86, *p* = 0.058) and mean distance between the animals (*F*(1, 32) = 3.72, *p* = 0.063) were found. Also, HOUSING revealed significant differences. Isolated mice from both genotypes showed an increased time spent in passive social interaction (*F*(1, 32) = 14.61, *p* < 0.01, Fig. 1b), higher numbers in sniffing (*F*(1, 32) = 45.68, *p* < 0.001) and anogenital sniffing behavior (*F*(1, 32) = 4.81, *p* < 0.05), lower rearing activity (*F*(1, 32) = 12.70, *p* < 0.01, Fig. 1c), increased grooming (*F*(1, 32) = 13.73, *p* < 0.01), and a higher number of aggressive contacts (*F*(1, 32) = 6.92, *p* < 0.05). Post hoc analyses for isolated *GAD67*^+*/GFP*^ mice, compared with isolated *GAD67*^+*/*+^ mice, showed a trend towards a lower time spent in social contact (*t* = − 2.02, *df* = 15, *p* = 0.062, Fig. 1a) and significantly less time spent in passive social interaction (*t* = − 2.32, *df* = 15, *p* < 0.05, Fig. 1b), indicating that post-weaning social isolation affects social behavior in *GAD67*^+*/GFP*^ mice. Group-housed *GAD67*^+*/GFP*^ mice, compared with group-housed *GAD67*^+*/*+^mice, displayed only an increased rearing activity (*t* = 2.88, *df* = 17, *p* = 0.01, Fig. 1c). Thus, male *GAD67*^+*/GFP*^ mice show a significant reduction in social interaction, particularly obvious in the socially isolated group. Further, isolated *GAD67*^+*/*+^mice, compared with group-housed *GAD67*^+*/*+^, spent significantly more time in passive social interaction (*t* = 3.86, *df* = 7.63, *p* < 0.01, Fig. 1b), showed an increased sniffing (*T* = 6.18, *df* = 16, *p* < 0.001) and grooming behavior (*T* = 2.45, *df* = 16, *p* < 0.05). Isolated *GAD67*^+*/GFP*^ mice, compared with group-housed *GAD67*^+*/GFP*^, presented a reduced rearing activity (*T* = -3.54, *df* = 16, *p* < 0.01, Fig. 1c), an increased sniffing (*t* = 3.84, *df* = 16, *p* < 0.01) and grooming behavior (*t* = 2.79, *df* = 16, *p* < 0.05). With regard to distance moved (Fig. 1d) and time spent in unprotected center zone of the open field, no significant main effects for GENOTYPE or HOUSING were found.

### Decreased social dominance in GAD67^+/GFP^ mice

GABA, dopamine, and serotonin under various conditions are associated with aggressive behavior (Miczek et al. 1994; Narvaes and Martins de Almeida 2014). Therefore, the social dominance tube test was performed by pairing group-housed or socially isolated male mice of both genotypes. We found that *GAD67*^+*/GFP*^ mice lost significantly more bouts, independent from housing condition (Fig. 1e, binary logistic model followed by Wald Chi-square test, GENOTYPE effect: Wald-*χ*^2^(1) = 7.18, Exp(*B*) = 0.39 (95% confidence interval (CI) = 0.20–0.78), *p* = 0.007; HOUSING effect: Wald-*χ*^2^(1) = 0.12, Exp(*B*) = 0.89 (95% CI = 0.45–1.75), *p* > 0.05; HOUSING and GENOTYPE interaction: Wald-*χ*^2^ (1) = 0.24, Exp(*B*) = 1.27 (95% CI = 0.49–3.30), *p* > 0.05, demonstrating that *GAD67*^+*/GFP*^ mice are less dominant than wild-type mice when paired against each other. Exploring group-housed mice, *GAD67*^+*/GFP*^ mice lost more bouts then *GAD67*^+*/*+^ mice (*χ*^2^ (1) = 4.11, *p* < 0.05, Fig. 1e). Additionally, this decrease in social dominance was found in socially isolated *GAD67*^+*/GFP*^ mice (*χ*^2^ (1) = 7.31, *p* < 0.01, Fig. 1e).

### Motor coordination is not impaired in *GAD67*^+*/GFP*^ mice

To determine possible alterations in motor coordination and balance of *GAD67*^+*/GFP* mice^, compared to *GAD67*^+*/*+^, the rotarod test was used. All mice showed learning of the rotarod and reached a stable level of performance within 3 days (Fig. 1f, g). A three-way repeated-measures ANOVA (within-subject factor: TRIAL, between-subject factors: HOUSING and GENOTYPE) revealed no significant interaction and no main effect for GENOTYPE (n.s.). HOUSING only showed a trend towards to an increase in rotarod performance for isolated mice (*F*(1, 44) = 3.64, *p* = 0.063).

### Increased depressive-like behavior in *GAD67*^+*/GFP*^ mice

To investigate the effects of GAD67 haplodeficiency and social isolation on depressive-like behavior male isolated and group-housed *GAD67*^+*/GFP*^ and *GAD67*^+*/*+^ mice were analyzed in the forced swim test (see Fig. 1h–j). A two-way multivariate ANOVA (between-subject factors: GENOTYPE and HOUSING) showed no significant interactions (n.s.). GENOTYPE revealed, that *GAD67*^+*/GFP*^ exhibit a significantly reduced latency to appearance of first immobility (*F*(1, 55) = 6.23, *p* < 0.05, Fig. 1h), increased time spent in immobility (*F*(1, 55) = 50.67, *p* < 0.001, Fig. 1i), reduced time spent swimming (*F*(1, 55) = 58.61, *p* < 0.001) and increased climbing behavior (*F*(1, 55) = 7.48, *p* < 0.01, Fig. 1j). HOUSING showed that isolated *GAD67*^+*/GFP*^ and *GAD67*^+*/*+^ mice had a significantly higher latency to appearance of first immobility period (Fig. 1h) compared with group-housed mice (*F*(1, 55) = 14.04, *p* < 0.001). Post hoc tests demonstrated a decreased latency to first immobility period (Fig. 1h) for isolated *GAD67*^+*/GFP*^ (*t* = − 2.79, *df* = 22.51, *p* < 0.05), compared with isolated *GAD67*^+*/*+^ mice. This difference was not found for animals held in groups (n.s.). Group-housed and socially isolated *GAD67*^+*/GFP*^ mice showed a comparable latency to first immobility period (n.s.). However, social isolation increased latency to first immobility in *GAD67*^+*/*+^ mice (*t* = 3.03, *df* = 22.90, *p* < 0.01, Fig. 1h). Most important, for time spent in immobility (Fig. 1i) post hoc analyses showed that isolated (*t* = 5.43, *df* = 32.59, *p* < 0.001), but also group housed (*t* = 5.26, *df* = 21.2, *p* < 0.001) *GAD67*^+*/GFP*^ spent significantly more time in immobility compared to *GAD67*^+*/*+^ mice, respectively. Social isolation did not affect time in immobility (Fig. 1i) of *GAD67*^+*/GFP*^ (n.s.) and *GAD67*^+*/*+^ mice (n.s.). For swimming, post hoc analyses revealed that isolated (*t* = − 5.6, *df* = 32.64, *p* < 0.001), and group-housed (*t* = − 5.98, *df* = 19.76, *p* < 0.001) *GAD67*^+*/GFP*^ mice spent less time in active swimming, compared to *GAD67*^+*/*+^ mice. Isolation rearing did not impair swimming activity of *GAD67*^+*/GFP*^ (n.s.) and *GAD67*^+*/*+^ mice (n.s.). With regard to climbing (Fig. 1j), post hoc tests revealed a significantly increased activity for group-housed *GAD67*^+*/GFP*^ (*t* = 3.09, *df* = 18.65, *p* < 0.01), compared with group-housed *GAD67*^+*/*+^ mice. Isolated *GAD67*^+*/*+^ mice showed a trend towards a decreased climbing activity compared with group-housed *GAD67*^+*/*+^ mice (*t* = 1.79, *df* = 23.95, *p* = 0.087). Thus, isolated and group-housed *GAD67*^+*/GFP*^ mice, respectively, show a higher depression like behavior compared with *GAD67*^+*/*+^ mice.

### Social isolation influences the activity of *GAD67*^+*/GFP*^ on the elevated plus maze

The effects of GAD67 haplodeficiency and isolation rearing on anxiety-related behavior were investigated on the elevated plus maze (EPM, see Fig. 1k–m). A two-way multivariate ANOVA revealed no significant interactions of HOUSING and GENOTYPE (n.s) or main effects of GENOTYPE (n.s). HOUSING did not affect the number of open arm entries (n.s.) or the time spent in open arms (n.s., Fig. 1k). However, significant effects of HOUSING were found for number of total arms entries (*F*(1, 58) = 5.67, *p* < 0.05, Fig. 1l), distance traveled (*F*(1, 58) = 6.62, *p* < 0.05, Fig. 1m) and closed arms entries (*F*(1, 58) = 5.28, *p* < 0.05). Also, the ratio of number of open arm entries to the number of total arm entries, a measure of anxiety-related behavior independent of locomotor activity, was calculated, but no HOUSING and GENOTYPE effects were found (n.s.). On closer examination of possible isolation rearing effects, isolated *GAD67*^+*/GFP*^, compared with group-housed *GAD67*^+*/GFP*^ mice, showed an increase of total arms entries (*t* = 2.59, *df* = 31, *p* < 0.05, Fig. 1l), distance traveled (*t* = 3.01, *df* = 31, *p* < 0.01, Fig. 1m), and closed arms entries (*t* = 2.55 *df* = 31, *p* < 0.05), whereas isolated and group-housed *GAD67*^+*/*+^ showed no significant housing differences (n.s.). Thus, as a sign of an increased locomotor activity on EPM, post-weaning social isolation results in significantly more arm entries and an increased distance traveled of isolated *GAD67*^+*/GFP*^ mice.

### No alteration in prepulse inhibition of acoustic startle response

**Fig. 2:**
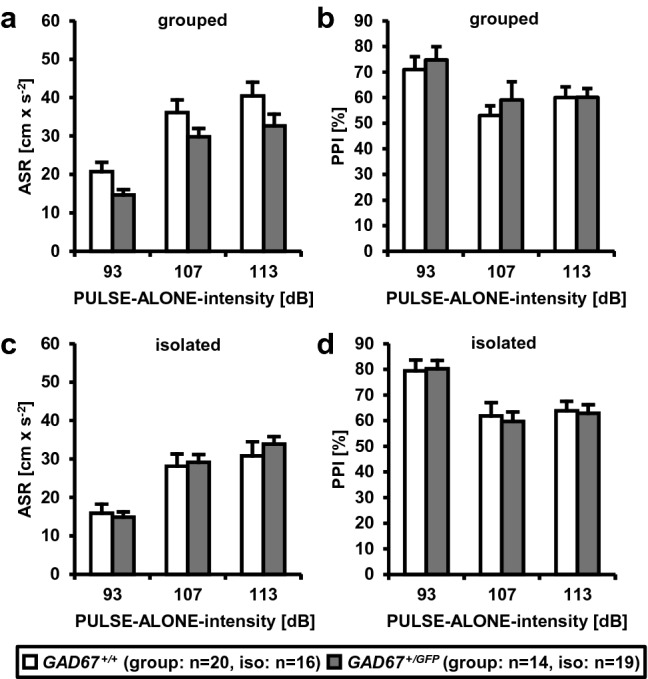
*GAD67*^+*/GFP*^ mice show no deficits in prepulse inhibition of acoustic startle reflex. **a**, **c** Acoustic startle response (ASR) and **b**, **d** prepulse inhibition (PPI) of group-housed and socially isolated *GAD67*^+*/*+^ and *GAD67*^+*/GFP*^ mice. No significant interactions or main effects were found, respectively. Data are presented as mean ± SEM. ASR and PPI were analyzed using three-way repeated-measures ANOVAs (within-subject factor: INTENSITY (three levels: 93, 107 and 113 dB), between-subject factors: GENOTYPE (two levels: *GAD67*^+*/*+^ and *GAD67*^+*/GFP*^) and HOUSING (two levels: group housed and isolated)

The acoustic startle response (ASR) and prepulse inhibition of acoustic startle (PPI) were analyzed at three different intensities (93, 107, 113 dB SPL) in male *GAD67*^+*/GFP*^ and *GAD67*^+*/*+^ mice kept either in groups or social isolation (see Table 2, Fig. 2a–d). The three-way repeated-measures ANOVAs (within-subject factor: INTENSITY, between-subject factors: HOUSING and GENOTYPE) revealed no significant interactions or main effects for ASR and PPI, respectively. Thus, a heterozygous deletion of the GAD67 gene and social isolation do not affect neither the startle reflex responses nor the degree of PPI.

### Tyrosine hydroxylase-IR neurons in substantia nigra/VTA

A hallmark of neuropsychiatric disorders is the disturbance of the dopaminergic system. Therefore, we analyzed *GAD67*^+*/GFP*^ and *GAD67*^+*/*+^ mice for differences in neuronal numbers, volumes and neuronal densities of TH-IR neurons in SN subregions SNC, SNR and SNL, respectively (see Table 3). Two-way repeated-measures ANOVAs (within-subject factor: REGION; between-subject factor: GENOTYPE) revealed no significant interactions or main effects (n.s.). Additionally, no genotype differences were found in neuron number, volume and neuron density of VTA (n.s.; Welch’s tests). Thus, *GAD67*^+*/GFP*^ and *GAD67*^+*/*+^ mice present no morphological differences in the investigated monoaminergic regions of the midbrain (see also supplementary Fig. S5).

**Table 3:** Quantification of TH-IR neurons and fiber densities in *GAD67*^+*/*+^ and *GAD67*^+*/GFP*^ mice

TH-IR neurons and fiber densities					Statistical analysis		
TH-IR neurons	Region	Layer	*GAD67*^+*/*+^ (*n* = 6)	*GAD67*^+*/GFP*^ (*n* = 6)	REGION/LAYER × GENOTYPE	Effect of GENOTYPE	Post hoc
Neuron number [*n*]	SNC		494.21 ± 28.55	465.96 ± 23.49			–
	SNR		41.26 ± 2.61	45.77 ± 5.74	*F*(1.04, 10.44) = 0.68, n.s	*F*(1, 10) = 0.44, n.s	–
	SNL		14.06 ± 1.10	11.28 ± 1.08			–
Volume [mm^3^]	SNC		0.08 ± 0.00	0.08 ± 0.01			–
	SNR		0.15 ± 0.01	0.14 ± 0.01	*F*(2, 20) = 2.61, n.s	*F*(1, 10) = 2.31, n.s	–
	SNL		0.01 ± 0.00	0.01 ± 0.00			–
Density [*n*/mm^3^]	SNC		6077.67 ± 239.37	6031.82 ± 214.57			–
	SNR		269.47 ± 11.50	334.70 ± 33.74	*F*(1.30, 13.03) = 0.12, n.s	*F*(1, 10) = 0.01, n.s	–
	SNL		1185.79 ± 61.12	1137.79 ± 120.15			–
Neuron number [*n*]	VTA		283.46 ± 26.88	281.23 ± 10.20	–	n.s. (Welch’s test)	
Volume [mm^3^]	VTA		0.07 ± 0.00	0.06 ± 0.00		n.s. (Welch’s test)	
Density [*n*/mm^3^]	VTA		3977.40 ± 259.93	4344.88 ± 114.01		n.s. (Welch’s test)	
**TH-IR fiber densities [μm/μm**^**3**^**]**							
Dorsal hippocampus	CA1	Or	0.06 ± 0.00	0.07 ± 0.00	*F*(2, 20) = 0.02, n.s	*F*(1, 10) = 20.85, ***p < 0.01***	***p < 0.001***
		Rad	0.08 ± 0.01	0.09 ± 0.01			***p < 0.05***
		LMol	0.09 ± 0.00	0.10 ± 0.01			***p < 0.05***
	CA3	Or	0.08 ± 0.01	0.09 ± 0.02	*F*(2, 20) = 0.30, n.s	*F*(1, 10) = 1.35, n.s	–
		Rad	0.11 ± 0.02	0.12 ± 0.02			–
		LMol	0.09 ± 0.01	0.09 ± 0.01			–
	DG	Mol	0.06 ± 0.00	0.08 ± 0.02	*F*(1, 10) = 0.27, n.s	*F*(1, 10) = 6.29, **p < 0.05**	n.s
		ML	0.09 ± 0.02	0.10 ± 0.01			n.s
Amygdala	BLA		0.36 ± 0.01	0.37 ± 0.01	*F*(3, 30) = 0.56, n.s	*F*(1, 10) = 0.11, n.s	–
	CeC		0.32 ± 0.02	0.30 ± 0.01			–
	CeM		0.51 ± 0.01	0.50 ± 0.01			–
	La		0.25 ± 0.01	0.25 ± 0.01			–

Coronal brains slices of *GAD67*^+*/*+^ and *GAD67*^+*/GFP*^ mice were investigated for densities of tyrosine hydroxylase (TH)-immunoreactive (IR) neurons in subregions of substantia nigra (SN) and ventral tegmental area (VTA) and densities of TH-IR fibers in the dorsal hippocampus and amygdala. Values represent mean ± SEM. Differences between *GAD67*^+*/*+^ and *GAD67*^+*/GFP*^ littermates were evaluated using repeated-measures ANOVAs with LAYER or REGION (two to four levels, depending on the investigated brain region) as the within-subject factors and GENOTYPE (two levels: *GAD67*^+*/*+^ and *GAD67*^+*/GFP*^) as the between-subject factor. The interactions and main effects of GENOTYPE are shown. Post hoc analyses were performed using unpaired *t* tests (Welch’s test) with Bonferroni–Holm adjustment. Neuron densities in the VTA were analyzed using unpaired *t* test (Welch’s test). For abbreviations, see list

### Tyrosine hydroxylase-positive fibers in dorsal hippocampus and amygdala

**Fig. 3:**
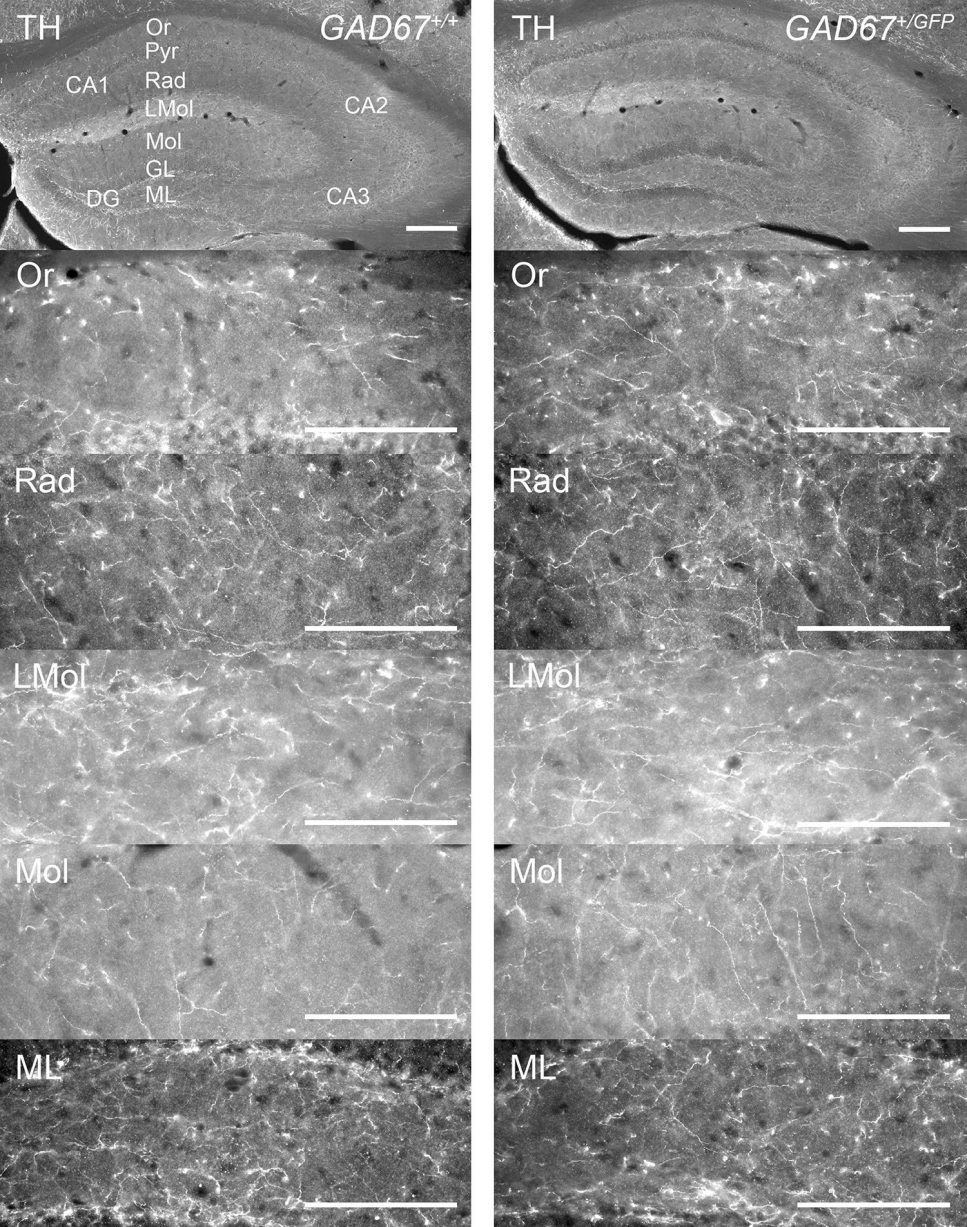
Distribution of TH-IR fibers in dorsal hippocampus of *GAD67*^+*/*+^ and *GAD67*^+*/GFP*^ mice. Microphotographs of coronal sections on the top give an overview. From top down images from CA1 and DG layers (CA1: Or, Rad, LMol; DG: Mol, ML) of each genotype are shown. The exemplary images are taken from the same mouse, respectively. Scale bar, 100 µm. For abbreviations, see list

**Fig. 4:**
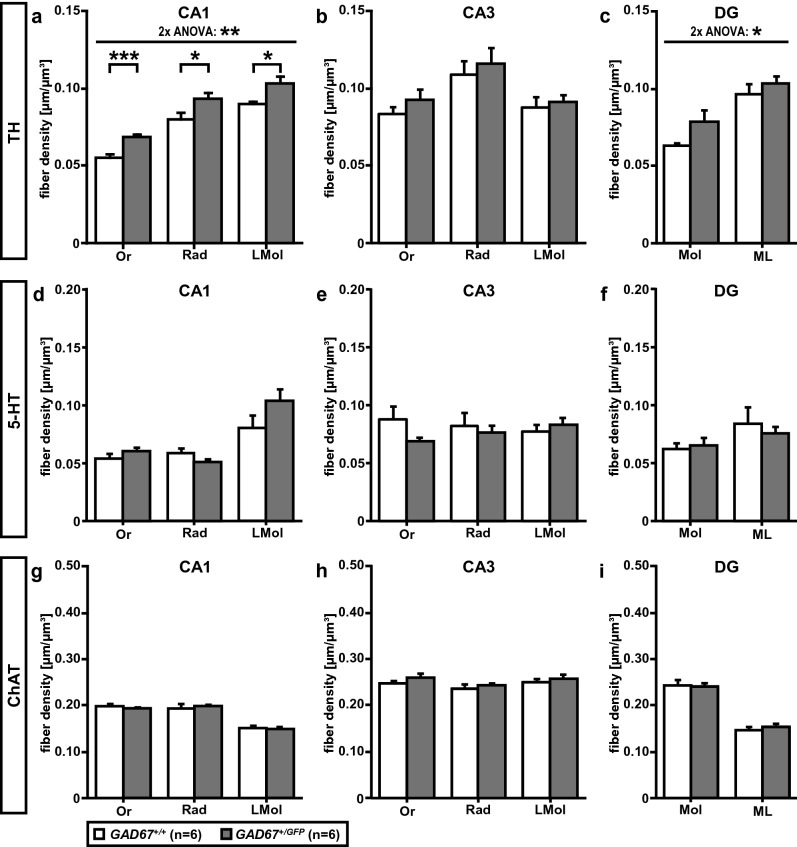
Tyrosine hydroxylase (TH)-, serotonin (5-HT)- and choline acetyltransferase (ChAT)-positive fiber densities in the dorsal hippocampal formation of *GAD67*^+*/*+^ and *GAD67*^+*/GFP*^ mice. **a**–**c** For TH-IR fiber densities a significant main effect of GENOTYPE was found in **a** CA1 and **c** DG, but not **b** CA3. **a** For *GAD67*^+*/GFP*^ the post hoc tests showed a significantly higher density of TH-IR fibers in all investigated layers of CA1 (Or, Rad and LMol). **c** For DG the alpha adjusted post hoc tests did not reach statistical significance. Investigating **d**–**f** 5-HT- and **g**–**i** ChAT-positive fiber densities, no differences between *GAD67*^+*/*+^ and *GAD67*^+*/GFP*^ mice (n.s.) were found. Statistics: data are reported as mean ± SEM. Two-way repeated measures ANOVAs were performed using LAYER (three levels in dorsal CA1 and CA3: Or, Rad, Lm or two levels in DG: Mol, Hil) as within-subject factor and GENOTYPE (two levels: *GAD67*^+*/*+^ and *GAD67*^+*/GFP*^) as between-subject factor. Post hoc analyses were carried out using unpaired t tests with Bonferroni–Holm adjustment. For abbreviations, see list. *p < 0.05; **p < 0.01; ***p < 0.001

To study the impact of GAD67 haplodeficiency on monoaminergic innervation of dorsal hippocampus, we estimated the densities of TH-IR fibers in three layers of CA1, CA3 (Or, Rad, LMol) and two layers of DG (Mol, ML), respectively (Table 3; Figs. 3, 4a–c). For CA1 (Fig. 4a) a two-way repeated-measures ANOVA showed no interaction of LAYER × GENOTYPE (n.s.) but a significant main effect of GENOTYPE (*F*(1, 10) = 20.85, *p* < 0.01). Post hoc analyses showed that *GAD67*^+*/GFP*^, compared to *GAD67*^+*/*+^ mice, have a significantly higher density of TH-IR fibers in Or (*t* = 6.48, *df* = 8.42, *p* < 0.001), Rad (*t* = 2.54, *df* = 9.90, *p* < 0.05), and LMol (*t* = 3.02, *df* = 5.95, *p* < 0.05) of CA1. With reference to dorsal CA3 (Fig. 4b) a two-way repeated-measures ANOVA showed no significant interactions or main effects (n.s.). Investigating the dorsal DG (Fig. 4c), no interaction (n.s.) but a significant main effect of GENOTYPE (*F*(1, 10) = 6.29, *p* < 0.05) was found. However, the post hoc analyses did not reach statistical significance (n.s.). In the amygdala TH-IR fiber densities were estimated in: LA, BLA, CeC, and CeM (Table 3) as described above. The two-way repeated-measures ANOVA revealed no significant interaction or main effect (n.s.). Thus, *GAD67*^+*/GFP*^ and *GAD67*^+*/*+^ mice demonstrate a higher TH-IR fiber density in dorsal hippocampal CA1, but no differences in other investigated subfields of dorsal hippocampus and amygdala.

### Serotonin-IR neurons in the raphe nuclei

Absolute neuron numbers, volumes, and neuronal densities of 5-HT-IR neurons were estimated in different raphe nuclei: DR, MnR and PMnR (Table 4). The two-way repeated-measures ANOVAs (within-subject factor: REGION; between-subject factor: GENOTYPE) revealed no significant interaction or main effects (n.s.). *GAD67*^+*/GFP*^ and *GAD67*^+*/*+^ mice show no differences in the investigated raphe nuclei (see also supplementary Fig. S6).

**Table 4:** Quantification of 5-HT-IR neurons and fiber densities in *GAD67*^+*/*+^ and *GAD67*^+*/GFP*^ mice

5-HT-IR neurons and fiber densities					Statistical analysis		
5-HT-IR neurons	Region	Layer	*GAD67*^+*/*+^ (*n* = 6)	*GAD67*^+*/GFP*^ (*n* = 6)	REGION/LAYER × GENOTYPE	Effect of GENOTYPE	Post hoc
Neuron number [*n*]	DR		1812.13 ± 100.10	1841.95 ± 49.85			–
	MnR		368.19 ± 17.21	344.92 ± 23.38	*F*(1.06, 10.60) = 0.20, n.s	*F*(1, 10) = 0.01, n.s	–
	PMnR		153.17 ± 6.13	132.97 ± 9.52			–
Volume [mm^3^]	DR		0.09 ± 0.00	0.10 ± 0.00			–
	MnR		0.03 ± 0.00	0.03 ± 0.00	*F*(2, 20) = 1.70, n.s	*F*(1, 10) = 0.08, n.s	–
	PMnR		0.06 ± 0.00	0.06 ± 0.00			–
Density [n/mm^3^]	DR		19643.03 ± 846.27	19120.55 ± 467.40			–
	MnR		12348.37 ± 819.33	11293.96 ± 791.83	*F*(2, 20) = 0.32, n.s	*F*(1, 10) = 0.91, n.s	–
	PMnR		2615.53 ± 118.65	2370.43 ± 197.58			–
**5-HT-IR fiber densities [μm/μm]**^3^							
Dorsal hippocampus	CA1	Or	0.05 ± 0.01	0.06 ± 0.00	*F*(1.19, 11.90) = 3.25, n.s	*F*(1, 10) = 2.55, n.s	–
		Rad	0.06 ± 0.01	0.05 ± 0.00			–
		LMol	0.08 ± 0.02	0.10 ± 0.02			–
	CA3	Or	0.09 ± 0.03	0.07 ± 0.01	*F*(1.33, 13.33) = 4.34, ***p < 0.05***	*F*(1, 10) = 0.11, n.s	–
		Rad	0.08 ± 0.03	0.08 ± 0.01			–
		LMol	0.07 ± 0.02	0.08 ± 0.01			–
	DG	Mol	0.06 ± 0.01	0.07 ± 0.02	*F*(1, 10) = 0.95, n.s	*F*(1, 10) = 0.00, n.s	–
		ML	0.08 ± 0.03	0.08 ± 0.01			–
Amygdala	BLA		0.21 ± 0.01	0.20 ± 0.01	*F*(3, 30) = 1.26, n.s	*F*(1, 10) = 0.02, n.s	–
	CeC		0.19 ± 0.01	0.18 ± 0.01			–
	CeM		0.21 ± 0.00	0.20 ± 0.01			–
	La		0.18 ± 0.02	0.20 ± 0.01			–

*GAD67*^+*/*+^ and *GAD67*^+*/GFP*^ mice were investigated for differences in densities of serotonin (5-HT)-immunoreactive (IR) neurons in the raphe nuclei and densities of 5-HT-IR fibers in dorsal hippocampus and amygdala. Values represent mean ± SEM. Differences between in *GAD67*^+*/*+^ and *GAD67*^+*/GFP*^ littermates were evaluated using repeated-measures ANOVAs with LAYER or  REGION (two to four levels, depending on the investigated brain region) as the within-subject factors and GENOTYPE (two levels: *GAD67*^+*/*+^*and GAD67*^+*/GFP*^) as the between-subject factor. The interactions and main effects of GENOTYPE are shown. Post hoc analyses were performed using unpaired *t* tests (Welch’s test) with Bonferroni–Holm adjustment. For abbreviations, see list

### Serotonin-positive fibers in dorsal hippocampus and amygdala

**Fig. 5:**
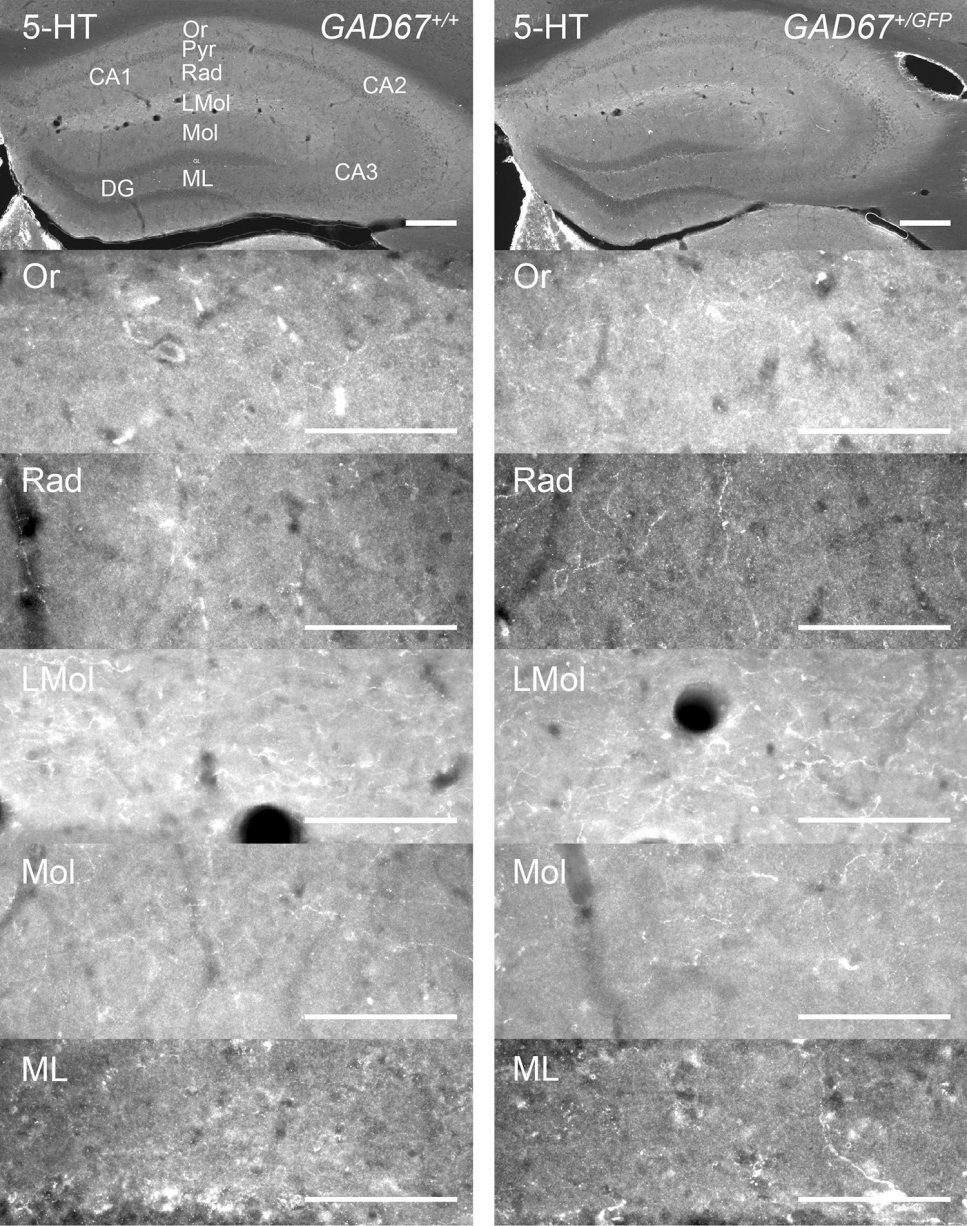
Microphotographs of coronal sections showing the distribution of 5-HT-IR fibers in dorsal hippocampus of *GAD67*^+*/*+^ and *GAD67*^+*/GFP*^ mice. Images on the top give an overview. From top down images from CA1 and DG layers (CA1: Or, Rad, LMol; DG: Mol, ML) of genotype line are shown. The exemplary images are taken from the same mouse, respectively. Scale bar, 100 µm. For abbreviations, see list

We further analyzed if *GAD67*^+*/GFP*^ and *GAD67*^+*/*+^ mice show differences in densities of 5-HT-IR fibers in layers of dorsal hippocampal CA1, CA3 (Or, Rad, LMol) and DG (Mol, ML), respectively (Table 4; Figs. 4d–f and 5). For CA1 and DG (Fig. 4d, f) two-way repeated-measures ANOVAs (within-subject factor: LAYER; between-subject factor: GENOTYPE) revealed no significant interactions or main effects (n.s.). With reference to CA3 (Fig. 4e) the two-way repeated-measures ANOVA showed a significant interaction of LAYER and GENOTYPE (*F*(1.33, 13.33) = 4.34, *p* < 0.05) but no main effect for GENOTYPE (n.s.). Additionally, in the amygdala (Table 4) no significant interaction or main effect was found (ANOVA, n.s). Therefore, *GAD67*^+*/GFP*^ and *GAD67*^+*/*+^ mice show comparable 5-HT-IR fiber densities in the investigated subregions of dorsal hippocampus and amygdala.

### Choline acetyltransferase-IR neurons in septal region

To determine if *GAD67*^+*/GFP*^ and *GAD67*^+*/*+^ littermates show differences in markers of the cholinergic system, we investigated both genotypes for neuronal numbers, volumes and neuronal densities of ChAT-positive neurons in medial (MS) and lateral (LS) septal region (see Table 5). For neuron number a two-way repeated-measures ANOVA (within-subject factor: REGION; between-subject factor: GENOTYPE) showed a significant interaction of REGION and GENOTYPE (*F*(1, 10) = 9.32, *p* < 0.05), but no significant effect of GENOTYPE (n.s.). Further, we examined both genotypes for differences in subregion volume and neuronal densities of MS and LS. Two-way repeated-measures ANOVAs (within-subject factor: REGION; between-subject factor: GENOTYPE) showed no significant interactions or main effects (n.s.), respectively. Therefore, *GAD67*^+*/GFP*^ and *GAD67*^+*/*+^ littermates show comparable cholinergic markers in the investigated septal subregions (see also supplementary Fig. S7).

**Table 5:** Quantification of ChAT-IR neurons and fiber densities in *GAD67*^+*/*+^ and *GAD67*^+*/GFP*^ mice

ChAT-IR neurons and fiber densities					Statistical analysis		
ChAT-IR neurons	Region	Layer	*GAD67*^+*/*+^ (*n* = 6)	*GAD67*^+*/GFP*^ (*n* = 6)	REGION/LAYER × GENOTYPE	Effect of GENOTYPE	Post hoc
Neuron number [*n*]	MS		968.16 ± 61.90	1185.23 ± 54.63	*F*(1, 10) = 9.32, ***p < 0,05***	*F*(1, 10) = 2.57, n.s	–
	LS		755.67 ± 49.84	745.63 ± 42.49			–
Volume [mm^3^]	MS		0.19 ± 0.02	0.22 ± 0.01	*F*(1, 10) = 0.32, n.s	*F*(1, 10) = 1.51, n.s	–
	LS		0.27 ± 0.03	0.30 ± 0.02			–
Density [n/mm^3^]	MS		5312.56 ± 369.73	5331.96 ± 219.38	*F*(1, 10) = 0.56, n.s	*F*(1, 10) = 0.17, n.s	–
	LS		2857.53 ± 221.14	2576.45 ± 225.76			–
**ChAT-IR fiber densities [μm/μm]**^3^							
Dorsal hippocampus	CA1	Or	0.20 ± 0.01	0.19 ± 0.01	*F*(2, 20) = 0.89, n.s	*F*(1, 10) = 0.04, n.s	–
		Rad	0.19 ± 0.02	0.20 ± 0.01			–
		LMol	0.15 ± 0.01	0.15 ± 0.01			–
	CA3	Or	0.25 ± 0.01	0.26 ± 0.02	*F*(2, 20) = 0.49, n.s	*F*(1, 10) = 1.61, n.s	–
		Rad	0.24 ± 0.01	0.24 ± 0.01			–
		LMol	0.25 ± 0.01	0.26 ± 0.02			–
	DG	Mol	0.24 ± 0.03	0.24 ± 0.01	*F*(1, 10) = 0.56, n.s	*F*(1, 10) = 0.00, n.s	–
		ML	0.15 ± 0.02	0.15 ± 0.02			–
Amygdala	BLA		0.59 ± 0.26	0.58 ± 0.12	*F*(3, 30) = 0.22, n.s	*F*(1, 10) = 0.15, n.s	–
	CeC		0.20 ± 0.01	0.19 ± 0.02			–
	CeM		0.38 ± 0.03	0.35 ± 0.03			–
	La		0.56 ± 0.25	0.55 ± 0.18			–

*GAD67*^+*/*+^ and *GAD67*^+*/GFP*^ mice were investigated for differences in densities of choline acetyltransferase (Chat)-IR neurons in the septal area and densities of Chat-IR fibers in dorsal hippocampus and amygdala. Values represent mean ± SEM. Differences between *GAD67*^+*/*+^ and *GAD67*^+*/GFP*^ littermates were evaluated using repeated-measures ANOVAs with LAYER or REGION (two to four levels, depending on the investigated brain region) as the within-subject factors and GENOTYPE (two levels: *GAD67*^+*/*+^ and *GAD67*^+*/GFP*^) as the between-subject factor. The interactions and main effects of GENOTYPE are shown. Post hoc analyses were performed using unpaired *t* tests (Welch’s test) with Bonferroni–Holm adjustment. For abbreviations, see list

### Choline acetyltransferase-positive fibers in dorsal hippocampus and amygdala

**Fig. 6:**
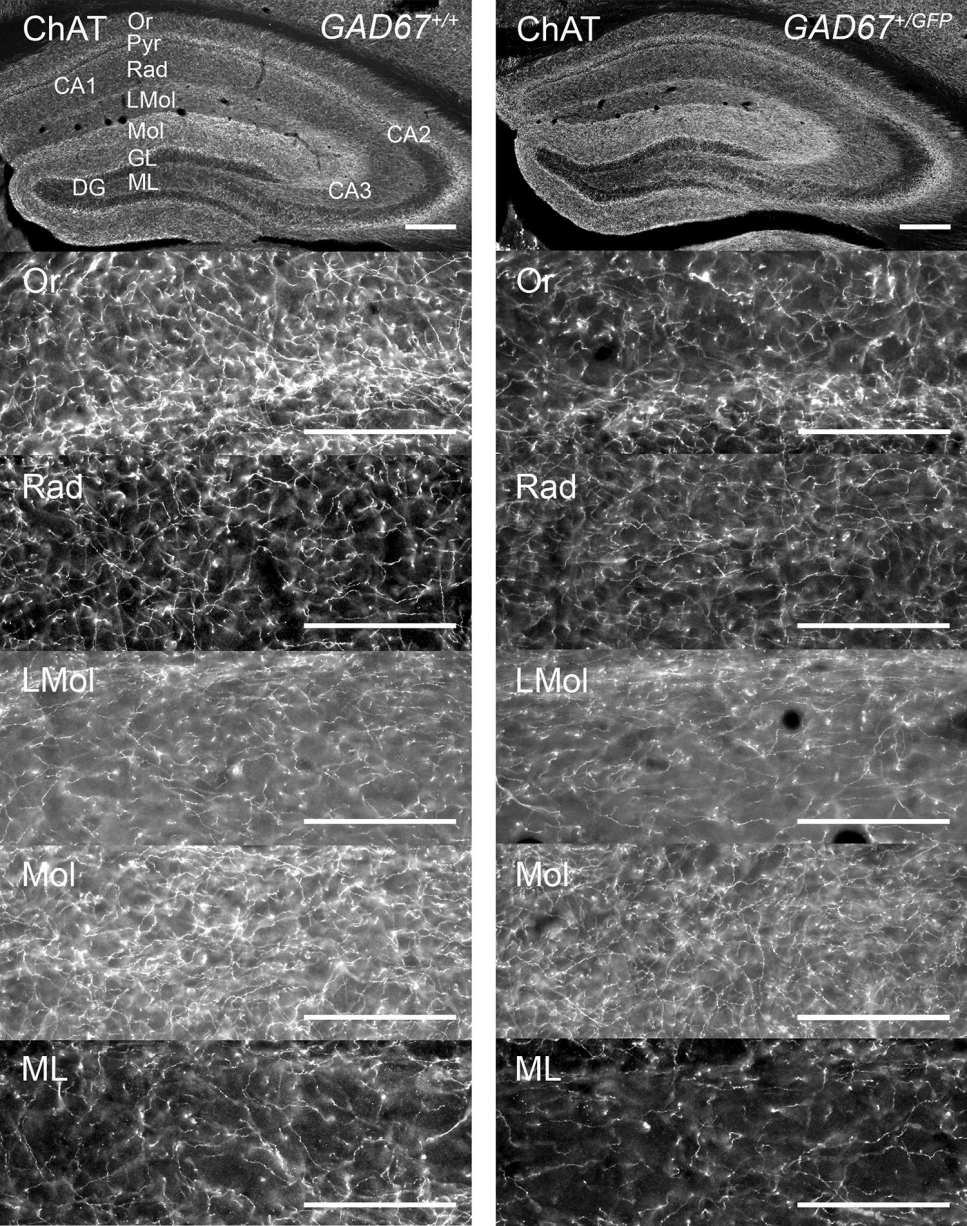
Distribution of ChAT-IR fibers in dorsal hippocampus of *GAD67*^+*/*+^ and *GAD67*^+*/GFP*^ mice. Microphotographs of coronal sections on the top give an overview. From top down images from layers (CA1: Or, Rad, LMol; DG: Mol, ML) of each genotype are shown. The exemplary images are taken from the same mouse, respectively. Scale bar, 100 µm. For abbreviations, see list

To investigate the cholinergic fiber systems of both genotypes, we estimated the densities of ChAT-IR fibers in dorsal hippocampal layers of CA1, CA3 (Or, Rad, LMol) and DG (Mol, ML), respectively (Table 5, Figs. 4g–i, 6). However, the two-way repeated-measures ANOVAs (within-subject factor: LAYER; between-subject factor: GENOTYPE) revealed no interactions or main effects (n.s), respectively (Fig. 4g–i). Additionally, in the amygdala (Table 5) no significant interactions or main effect (ANOVA, n.s.) were found. Thus, *GAD67*^+*/GFP*^ and *GAD67*^+*/*+^ mice show no differences in ChAT-IR fiber densities in the investigated subregions of dorsal hippocampus and amygdala.

## Discussion

In this study, we address the potential contribution of deficits in GAD67-mediated GABA synthesis in mice to behavioral and morphological phenotypes reminiscent of schizophrenia pathology. Our data reveal a profound alteration in *GAD67*^+*/GFP*^ mice in the socio-emotional domain, including an altered vulnerability to social deprivation and a selective catecholaminergic, presumably dopaminergic, hyperinnervation of area CA1 in the dorsal hippocampus.

### Social behavior, locomotor activity, anxiety and sensorimotor gating in *GAD67*^+*/GFP*^ mice

Negative symptoms like social withdrawal, avolition and depressive-like behavior are common patterns of human schizophrenia (Samsom and Wong 2015) and also apparent in animal models (Ellenbroek and Cools 2000; Matrisciano et al. 2013). Complex social behaviors and emotional processing such as anxiety are depending on different cerebral cortical areas, the hippocampus and the amygdala (Bicks et al. 2015). On the other hand, motivation and execution of motor plans are processed along parallel cortico-striatal-thalamic-cortical loops (Haber 2003). Alterations in those complex circuits could affect social behavior, locomotor activity, anxiety and other behavioral traits (Guillin et al. 2007). The social interaction test in the open field allows analyzing spontaneous induced behavior largely independent from external influences (File and Seth 2003). Our behavioral analysis showed that *GAD67*^+*/GFP*^ mice spent less time in social contact, marker of a profound disturbance in social interaction. Importantly, this alteration was independent from general motor activity, represented by a comparable distance moved of both genotypes in the open field. Our findings are in accordance with data from our previous study investigating sociability in *GAD67*^+*/GFP*^ mice using the three-chamber social preference test (Sandhu et al. 2014). Additionally, *GAD67*^+*/GFP*^ and *GAD67*^+*/*+^ mice where tested in the tube test for social dominance. This test was originally developed to analyze social hierarchy in mice through the measurement of aggression (Lindzey et al. 1961), which is known to be modulated by GABAergic function (Miczek et al. 2003). Independent from grouped and isolated housing conditions, *GAD67*^+*/GFP*^ retreated more frequently than *GAD67*^+*/*+^ mice suggesting a reduced social dominance. This finding is supported by data from our previous study, showing decreased aggressive like behavior of *GAD67*^+*/GFP*^ mice in the resident intruder test (Sandhu et al. 2014). However, we found no genotype differences in the number of aggressive contacts in the social interaction test, which may reflect different forms of social behavior and aggression in these two tasks (Nelson and Chiavegatto 2000).

Social behavior in rodents is highly complex and greatly depends on olfaction (Schultz and Tapp 1973). In our previous study, we found that male *GAD67*^+*/GFP*^ mice show a reduced preference for female mice and a reduced sensitivity to social and non-social odors, maybe reflecting an altered olfactory sense. As demonstrated by expression of the immediate early gene c-Fos we found an unaltered activation of the olfactory bulb in *GAD67*^+*/GFP*^ mice, but decreased neuronal activation in amygdala, bed nucleus of stria terminalis, medial preoptic area and lateral septum. It can be assumed, that *GAD67*^+*/GFP*^ mice show a disturbance in detection or, more importantly, processing of olfactory stimuli in downstream circuits relevant for social behavior (Sandhu et al. 2014). *GAD67*^+*/GFP*^ mice additionally showed altered non-social behavior, an increased rearing activity, in the social interaction test. This could be result of an impaired exploratory motor behavior and habituation of exploration in a novel environment, also reported for the DISC1 animal model of schizophrenia (Walsh et al. 2012). The social interaction test in the open field additionally allowed us to investigate the effects of post-weaning isolation rearing. Isolated male mice of both genotypes showed increased investigation of the unfamiliar test partner shown by higher numbers in sniffing, anogenital sniffing and aggressive contacts. This could be more result of the social isolation rearing (Shoji and Mizoguchi 2011) rather than of GAD67 haplodeficiency. Isolated mice also displayed an increased passive social behavior, which is seen when animals are sitting or lying close to each other, but without direct interaction (Sams-Dodd 1995). Additionally, we found lower rearing activity and increased repetitive self-grooming in isolated *GAD67*^+*/GFP*^ and *GAD67*^+*/*+^ mice. Our current results reveal that independent from genotype social isolation rearing increases stress and additionally alters specific aspects of explorative and social behavior. The latter is supported by the finding, that GAD67 haplodeficiency itself provokes higher corticosterone levels and enhances maternal and fetal stress vulnerability (Uchida et al. 2011). However, it is important to mention that not only genetic manipulations or social isolation stress could compromise the behavioral phenotype of an animal model. Additionally, the choice of the genetic background strain might influence the outcome of different tests paradigms. This is important, since it was shown, that the genetic background strain influences locomotor activity and anxiety like behavior (Bothe et al. 2005; Voikar et al. 2005).

Negative symptoms of schizophrenia-like poor social drive are commonly associated with depressive-like behavior (Häfner et al. 1999). To analyze depressive-like behavior, we investigated *GAD67*^+*/GFP*^ and *GAD67*^+*/*+^ mice in the forced swim test (FST) which is based on the assumption that immobility reflects a measure of behavioral despair (Borsini and Meli 1988). *GAD67*^+*/GFP*^ mice showed increased time spent in immobility, indicating an increase of depressive-like behavior. It was shown that the administration of low doses GABA or GABA agonists can ameliorate forced swimming induced depressive-like behavior and are able to potentiate the effect of antidepressants (Borsini et al. 1988; Aley and Kulkarni 1989). On the other hand, GABA antagonists like picrotoxin increase the immobility in FST (Poncelet et al. 1987). Therefore, we suggest GAD67 haplodeficiency provokes depressive-like behavior as found in neuropsychiatric diseases. Social isolation only resulted in higher latency to the first immobility period but did not affect immobility in the FST in general. This is in line with studies (Yates et al. 1991; Hall et al. 1998; Simpson et al. 2012) showing that social isolation in rodents has no effect on immobility in the FST or can promote despair-like immobility only when isolated in a short period of time during early brain development (17–21 day-old animals). Interestingly, group-housed *GAD67*^+*/GFP*^, compared to *GAD67*^+*/*+^ mice, showed an increased climbing behavior in the FST, which is reported to be a predictor of an increased motor activity in this test (Lino-de-Oliveira et al. 2005; Vieira et al. 2008). Therefore, we suggest that *GAD67*^+*/GFP*^ show an increased motor activity in FST, possible correlate of the positive symptom domain of schizophrenia (Jones et al. 2011).

Comorbid anxiety disorders are present in more than one third of patients with schizophrenia (Pokos and Castle 2006). The elevated plus maze (EPM) is commonly used as a behavioral assay to study anxiety like behavior in animal models (Walf and Frye 2007). However, our results reveal no differences in time spent in the open arms or locomotor activity between *GAD67*^+*/GFP*^ and *GAD67*^+*/*+^ mice. This is further supported the study of Smith (2018) reporting no differences in anxiety related behavior of *GAD67*^+*/GFP*^ on EPM. Modulation of the GABAergic system was shown to affect anxiety like behavior on EPM. GABA_A_ receptor agonists like diazepam or chlordiazepoxide, increase the proportion of time spent in open arms on EPM, whereas GABA_A_-receptor antagonists like picrotoxin reduce this measure (Lister 1987; Rodgers et al. 1992). It is possible that the decrease in GAD67 is not significant enough to provoke alterations of anxiety like behavior in *GAD67*^+*/GFP*^ mice. This assumption is partially supported by the finding, that *GAD67*^+/GFP^ mice showed no alterations in exploring the unprotected center area in social interaction task in the open field. Therefore, GAD67 haplodeficiency alone or in combination with social isolation rearing did not affect anxiety related behavior of *GAD67*^+*/GFP*^ mice, as demonstrated in the EPM and open-field test (Sandhu et al. 2014; Smith 2018). Thus, *GAD67*^+*/GFP*^ mice may not be a suitable model to investigate the anxiety-related symptom domain of neuropsychiatric disorders.

Reductions of GAD67 and PARV in mouse cerebral cortex and hippocampus are associated with novelty-induced hyperlocomotion (Belforte et al. 2010) considered to be a sign of schizophrenia-like behavior in human and mutant mice (Laviola et al. 2006). However, spontaneous locomotor activity and motor coordination in social interaction and rotarod test were not affected in *GAD67*^+*/GFP*^ mice, indicating preserved motor functions. Our findings are supported in part by the work of Smith (2018). This study also showed no genotype differences in EPM test, but showed a mild hyperactivity of *GAD67*^+*/GFP*^ mice in the open field may be result of a longer duration of testing. Social isolation stress induced higher locomotor activity and total arm entries of isolated *GAD67*^+*/GFP*^ mice on EPM, compared to group-housed *GAD67*^+*/GFP*^. This effect was not significant between isolated and group-housed *GAD67*^+*/*+^ mice. Since it was shown that postweaning social isolation affects GABAergic function (Hickey et al. 2012; Lim et al. 2012) and increases locomotor behavior on EPM (Abramov et al. 2004; Voikar et al. 2005), it is likely that the decrease of GAD67 alone is not significant enough to alter locomotor activity in *GAD67*^+*/GFP*^ mice. Therefore, we suggest that the additional exposure to social isolation stress as a “second hit” increases the vulnerability of a GAD67 haplodeficiency in *GAD67*^+*/GFP*^ mice, which results in impaired locomotor activity on the EPM. By contrast, spontaneous locomotor activity of isolated *GAD67*^+*/GFP*^ was not increased in the social interaction test in the open field.

It is unlikely that social interaction or aggression is reduced in *GAD67*^+*/GFP*^ mice because of a general deficit in sensorimotor function. Since several neuropsychiatric diseases including schizophrenia are accompanied by deficits in sensory information-processing (Braff et al. 2001), we analyzed *GAD67*^+*/GFP*^ mice and controls for their startle response (ASR) and prepulse inhibition (PPI). However, we found no genotype differences, which suggest that GAD67 haplodeficiency has no influence on ASR and PPI. By contrast, mice lacking GAD67 primarily in PARV-IR neurons show a reduction in PPI indicating the important role of these neurons in sensorimotor gating (Fujihara et al. 2015). It is possible, that the reduction of GAD67 and consequently GABA in *GAD67*^+*/GFP*^ mice is too small to cause sensorimotor gating deficits (Kolata et al. 2018). Since social isolation is used to model deficient sensorimotor gating in schizophrenia (Varty et al. 2006), we assumed that isolation rearing disrupts sensorimotor gating in our mice. Interestingly, neither *GAD67*^+*/GFP*^ nor *GAD67*^+*/*+^ mice showed ASR or PPI deficits as a result of post-weaning isolation housing. In consequence, we varied the parameters for PPI measurement. First, we increased the interval between prepulse and pulse to 400 ms and, second, varied the prepulse intensities (70, 75 and 80 dB SPL). Additionally, no genotype or housing differences were found (data not shown). We conclude that social isolation does not affect startle activity or sensorimotor gating in *GAD67*^+*/GFP*^ and *GAD67*^+*/*+^ mice, which is in line with other studies showing that social isolation during the critical developmental period has no effect on sensorimotor gating (Pietropaolo et al. 2008; Kulesskaya et al. 2011). However, PPI deficiency could be compromised by the duration of social isolation housing (Tueting et al. 2008), resulting in a comparatively strong decrease of GABA expression levels in *GAD67*^+*/*+^ control mice. Thus, long-term social isolation in adulthood could obscure the PPI deficits (Tueting et al. 2008) in *GAD67*^+*/GFP*^ mice compared to *GAD67*^+*/*+^, which could explain the negative findings in the present study.

### Tyrosine hydroxylase-IR neurons and fibers

Dopaminergic dysfunction in association with GAD67 deficiency is implicated in the pathophysiology of schizophrenia and associated with alterations in the hippocampus and amygdala (Laviolette 2007; Lodge and Grace 2011b; Brisch et al. 2014). Therefore, we were interested if GAD67 haplodeficiency is accompanied by alterations of the dopaminergic system. Tyrosine hydroxylase (TH) catalyzes the first and rate-determining step of the catecholamine biosynthesis and is expressed in all catecholaminergic neurons. Therefore, antibodies against TH-positive structures denote dopaminergic as well as noradrenergic neurons and fibers, but with regional differences (Asan 1993). We used tyrosine hydroxylase (TH)-immunoreactivity (IR) as a marker for dopamine since it is known to predominantly represent mesencephalic DAergic input in hippocampal CA1 of mice (Walling et al. 2012; for discussion see Nullmeier et al. 2014). In the amygdala, regionally different DAergic and noradrenergic innervation patterns are described and TH-IR fibers are found to be predominantly dopaminergic afferent fibers (Asan 1997). *GAD67*^+*/GFP*^ mice, compared to *GAD67*^+*/*+^, showed no difference in density of TH-IR neurons and volume of substantia nigra (SN) and ventral tegmental area (VTA). However, we found that *GAD67*^+*/GFP*^ mice exhibit a significantly higher density of TH-IR fibers in CA1 subfield of dorsal hippocampus, which can be interpreted as a hypercatecholaminergic, presumably hyperdopaminergic, innervation. Additionally, we investigated both genotypes for differences in TH-IR fiber density in the amygdala, but could not find any differences in the investigated subdivisions (Asan 1997).

It has been shown, that the dorsal hippocampus is crucial for spatial and long-term memories (Lodge and Grace 2011b; Brisch et al. 2014; Ragland et al. 2017) and modulates anxiogenic effects in the social interaction test (File et al. 1998). Additionally, dopamine D1-receptors in dorsal hippocampus were reported to mediate social learning and social behaviors in mice (Matta et al. 2017). Thus, it is possible, that the deficits in social interaction of *GAD67*^+*/GFP*^ mice are consequence of a dysfunction of the hippocampal dopaminergic system. However, we only found alterations in dorsal hippocampal CA1, but not in the amygdala and dopaminergic midbrain regions. It is likely, that the higher density of TH-IR fibers in CA1 of *GAD67*^+*/GFP*^ mice is induced locally by a GABAergic deficit in the hippocampus.

GAD67 reduction in the hippocampus is expected to lead to hyperactivity of midbrain dopamine neurons via a polysynaptic pathway (Kalkman and Loetscher 2003; Lodge and Grace 2011b). This appears to be mediated especially by a dysregulation of PARV-containing neurons (Lodge and Grace 2011a). A recent study, investigating GAD67 haplodeficiency showed that maternal stress postnatally decreases especially the density of PARV-positive GABAergic neurons in prefrontal cortex, hippocampus and somatosensory cortex of *GAD67*^+*/GFP*^ mice (Uchida et al. 2014). As a consequence, GABAergic dysfunction in hippocampus could lead to alterations in the dopaminergic pathways and subsequently social behavior, as found in the present study. Additionally, it can be assumed that alterations of local GABAergic circuits in VTA could provoke dopaminergic alterations. Thus, hyperdopaminergic innervation in association with a disturbance in social (Sandhu et al. 2014) and depressive-like behavior may mimic important aspects of neuropsychiatric disorders like schizophrenia (Lisman et al. 2008; Grace 2012).

### Serotonin-IR and choline acetyltransferase-IR neurons and fibers

The serotonergic and cholinergic systems are found to be involved in schizophrenia (Raedler et al. 2007; Geyer and Vollenweider 2008) and are in close interaction with the DAergic and GABAergic neurotransmitter systems (Kapur and Remington 1996; Scarr et al. 2013). Therefore, we were interested if GAD67 haplodeficiency is accompanied by alterations in both systems. However, *GAD67*^+*/GFP*^ and *GAD67*^+*/*+^ mice showed no differences in the density of the 5-HT positive neurons and volumes of the investigated raphe nuclei. The same applied to the density of ChAT-IR neurons and volumes of the septal nuclei. Additionally, we found no alterations in the densities of 5-HT-IR and ChAT-IR fibers in hippocampus and amygdala of *GAD67*^+*/GFP*^ mice. However, our data will only provide a descriptive morphological analysis and until now, impairments of the serotonergic or cholinergic system in *GAD67*^+*/GFP*^ mice have not been reported in the literature. Therefore, at the moment, it is difficult to make clear statements about the impact of GAD67 haplodeficiency on both neurotransmitter systems and vice versa.

### *GAD67*^+*/GFP*^ as a mouse model of schizophrenia and major depressive disorder

Schizophrenia and major depressive disorder (MDD) are considered two distinct neuropsychiatric diseases. However, there is an overlap between negative symptoms of schizophrenia and certain depressive symptoms like anhedonia, avolition and social withdrawal (Siris et al. 1988). Additionally, both disorders were shown to share alterations in biological markers of GABAergic transmission. Similar to schizophrenia, reductions of cortical GAD67 expression and alterations in GABA_A_ and GABA_B_ receptor levels were reported in MDD (Fatemi et al. 2005; Abdallah et al. 2015; Fogaca and Duman 2019). In contrast to schizophrenia, cortical GABA levels appear mostly decreased in MDD (Sanacora et al. 1999; Tayoshi et al. 2010; Luscher and Fuchs 2015). It is suggested that this reduction of GABA in MDD could not only result from decreased levels of GAD67, but could also result from a reduction in the density of specific GABA interneuron subclasses (reviewed in Fogaca and Duman 2019). This is supported by studies showing reduced volumes of prefrontal cortex and hippocampus (MacQueen et al. 2008; Savitz and Drevets 2009) and decreased cortical densities of calbindin- and somatostatin-positive GABAergic interneurons in MDD patients (Rajkowska et al. 2007; Sibille et al. 2011; Luscher and Fuchs 2015). PARV-positive interneurons play an important role in cognitive function, emotional response and social interaction (Ferguson and Gao 2018). However, the expression of parvalbumin (PARV) and density of PARV-positive interneurons mostly appear unaltered in MDD, which is in contrast to schizophrenia (reviewed in Fogaca and Duman 2019). On the other hand, rodent studies showed that the exposure to chronic stress or social isolation cause reductions of PARV-positive neurons in prefrontal cortex and hippocampus (Czeh et al. 2015, 2018; Todorovic et al. 2019). That maternal stress, in addition to a heterozygous deletion of GAD67, diminishes neurogenesis of GABAergic neurons was also shown in *GAD67*^+*/GFP*^ mice. Consequently, this postnatally results in a decreased density of PARV-positive interneurons in hippocampus, prefrontal and somatosensory cortex of *GAD67*^+*/GFP*^ mice (Uchida et al. 2014; Wang et al. 2018), similar to that found in human schizophrenia. Therefore, prenatal and social isolation stress could disturb the function of specific interneuron subpopulations and mechanism underlying the control of behaviors related to mood and emotion in neuropsychiatric disorders like schizophrenia and MDD. *GAD67*^+*/GFP*^ mice may provide a useful model for studying the impact of a heterozygous deletion of GAD67 on an enhanced vulnerability to prenatal and social isolation stress.

## Conclusion

Cerebral cortical and hippocampal GAD67 reductions are consistently reported in schizophrenia and other neuropsychiatric disorders (Akbarian et al. 1995; Guidotti et al. 2000; Benes and Berretta 2001; Hashimoto et al. 2003; Lewis et al. 2005). The present results suggest that GAD67 haplodeficiency in *GAD67*^+*/GFP*^ mice provokes profound disturbances in social behavior, social dominance and depressive-like behavior, which may reflect negative symptoms found in human schizophrenia and symptoms of MDD. In addition, our findings indicate that GAD67 haplodeficiency and social isolation stress may have additive influences on risks for developing schizophrenia or MDD (Fone and Porkess 2008). GAD67 haplodeficiency is further accompanied by a selectively increased density of TH-positive fibers in dorsal hippocampal CA1 suggesting an alteration of the dopaminergic system upstream to GABAergic dysfunction. *GAD67*^+*/GFP*^ mice are useful to model GABAergic hypofunction as disposition for the development of neuropsychiatric disorders like schizophrenia and MDD.

## Electronic supplementary material

Below is the link to the electronic supplementary material.

Supplementary file1 (DOCX 6469 kb)

## Abbreviations

5-HT: Serotonin
ASR: Acoustic startle response
BLA: Basolateral amygdala
CA1-3: Hippocampal cornu ammonis regions
CeC: Central amygdaloid nucleus, capsular part
CeM: Central amygdaloid nucleus, medial division
ChAT: Choline acetyltransferase
CI: Confidence interval
CPu: Caudate–putamen
DA: Dopamine
DG: Dentate gyrus
DR: Dorsal nucleus raphe
EPM: Elevated plus maze
Exp(B): Odds ratio
FST: Forced swim test
GAD: Glutamic acid decarboxylase
*GAD67*^+^^*/*^^+^: Wild type
*GAD67*^+^^*/GFP*^: *GAD67–GFP* Knock-in mice
GL: Granule cell layer of dentate gyrus
IR: Immunoreactivity
La: Lateral amygdala
LMol: Stratum lacunosum moleculare
LS: Medial septum
MDD: Major depressive disorder
ML: Multiform layer of dentate gyrus
MnR: Median raphe nucleus
Mol: Stratum moleculare of dentate gyrus
MS: Medial septum
NAc: Nucleus accumbens
NMDA-R: *N*-methyl-d-aspartate receptors
Or: Stratum oriens
PARV: Parvalbumin
PFC: Prefrontal cortex
PMnR: Paramedian raphe nucleus
PPI: Prepulse inhibition of acoustic startle response
Py: Stratum pyramidale
Rad: Stratum radiatum
SEM: Standard error of the mean
SN: Substantia nigra
SNC: Substantia nigra pars compacta
SNL: Substantia nigra pars lateralis
SNR: Substantia nigra pars reticulata
TH: Tyrosine hydroxylase
VTA: Ventral tegmental area
