# Supplementary material for: Glutamic acid decarboxylase 67 haplodeficiency in mice: consequences of postweaning social isolation on behavior and changes in brain neurochemical systems
Source: Brain Struct Funct. 2020 Jun 8;225(6):1719–42. doi: 10.1007/s00429-020-02087-6 (PMC7321906; doi:10.1007/s00429-020-02087-6)
Supplement: Supplementary file 1 — Supplementary file1 (DOCX 6469 kb) [file 429_2020_2087_MOESM1_ESM.docx]

Supplementary data for Brain Structure and Function:

Glutamic acid decarboxylase 67 haplodeficiency in mice: consequences of postweaning social isolation on behavior and changes in brain neurochemical systems

Nullmeier, S.^a^, Elmers, C.^b^, D’Hanis, W. ^b^, Sandhu, K. V.^c^, Stork, O^c,f^, Yanagawa Y.^d^, Panther, P.^e^, Schwegler, H.^b^

**Affiliations:**

^a^Institute of Molecular and Cellular Anatomy, Ulm University, Ulm, Germany

^b^Institute of Anatomy, Otto-von-Guericke University Magdeburg, Magdeburg, Germany

^c^Department of Genetics and Molecular Neurobiology, Institute of Biology, Otto-von-Guericke University Magdeburg, Magdeburg, Germany

^d^Department of Genetic and Behavioral Neuroscience, Gunma University Graduate School of Medicine, Maebashi, Gunma, Japan

^e^Department of Neurosurgery, Ulm University Medical Center, Ulm, Germany

^f^Center for Behavioral Brain Sciences, Universitätsplatz 2, 39106 Magdeburg, Germany

# Corresponding author

Sven Nullmeier, M.D.

Institute of Molecular and Cellular Anatomy, Ulm University

Albert-Einstein-Allee 11, D-89081 Ulm

Phone: +49-(0)731-500-23104

Fax : +49-(0)731-500-23102

E-mail: sven.nullmeier@uni.ulm.de

# Abbreviations

5-HT, serotonin; CA1-3, hippocampal Cornu Ammonis regions; ChAT, choline acetyltransferase; DA, dopamine; DG, dentate gyrus; DR, dorsal nucleus raphe; GAD, glutamic acid decarboxylase; *GAD67^+/+^*, wild type; *GAD67^+/GFP^*, *GAD67-GFP* knock in mice; GL, granule cell layer of dentate gyrus; HPC, hippocampus; IR, immunoreactivity; LMol, stratum lacunosum moleculare; LS, medial septum; ML, multiform layer of dentate gyrus; Mol, stratum moleculare of dentate gyrus; MS, medial septum; Or, stratum oriens; PFC, prefrontal cortex; Py, stratum pyramidale; Rad, stratum radiatum; SEM, standard error of the mean; SN, substantia nigra; SNC, substantia nigra pars compacta; SNL, substantia nigra pars lateralis; SNR, substantia nigra pars reticulata; TH, tyrosine hydroxylase; VTA, ventral tegmental area.

# Supplementary figures

**
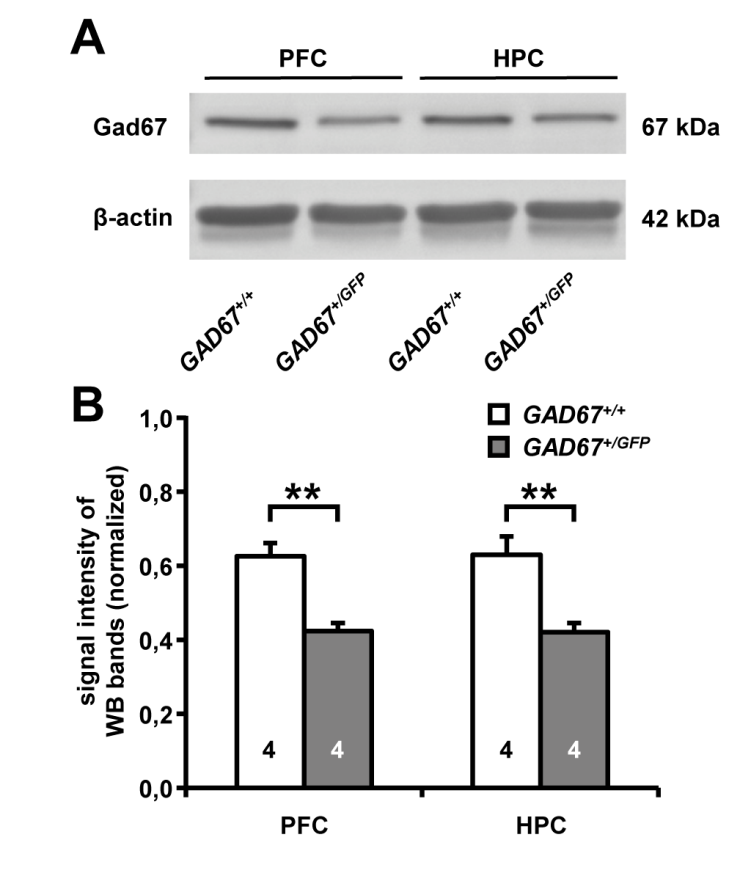
**

**Fig. S1.** Western blot analysis of GAD67 protein expression in prefrontal cortex and hippocampus of *GAD67^+/+^* and *GAD67^+/GFP^* mice. (a) Representative Western immunoblot bands illustrate the expression of GAD67 protein and that of housekeeping protein β-actin. (b) Densitometric analysis showed that GAD67 protein, normalized against β-actin, was decreased by 33% in prefrontal cortex (PFC) and by 32% in hippocampus (HPC) of *GAD67^+/GFP^* mice, compared to *GAD67^+/+^* mice. Data are presented as mean ± SEM. Western blot analysis was performed using unpaired *t* tests, respectively. ***p<0.01*

**
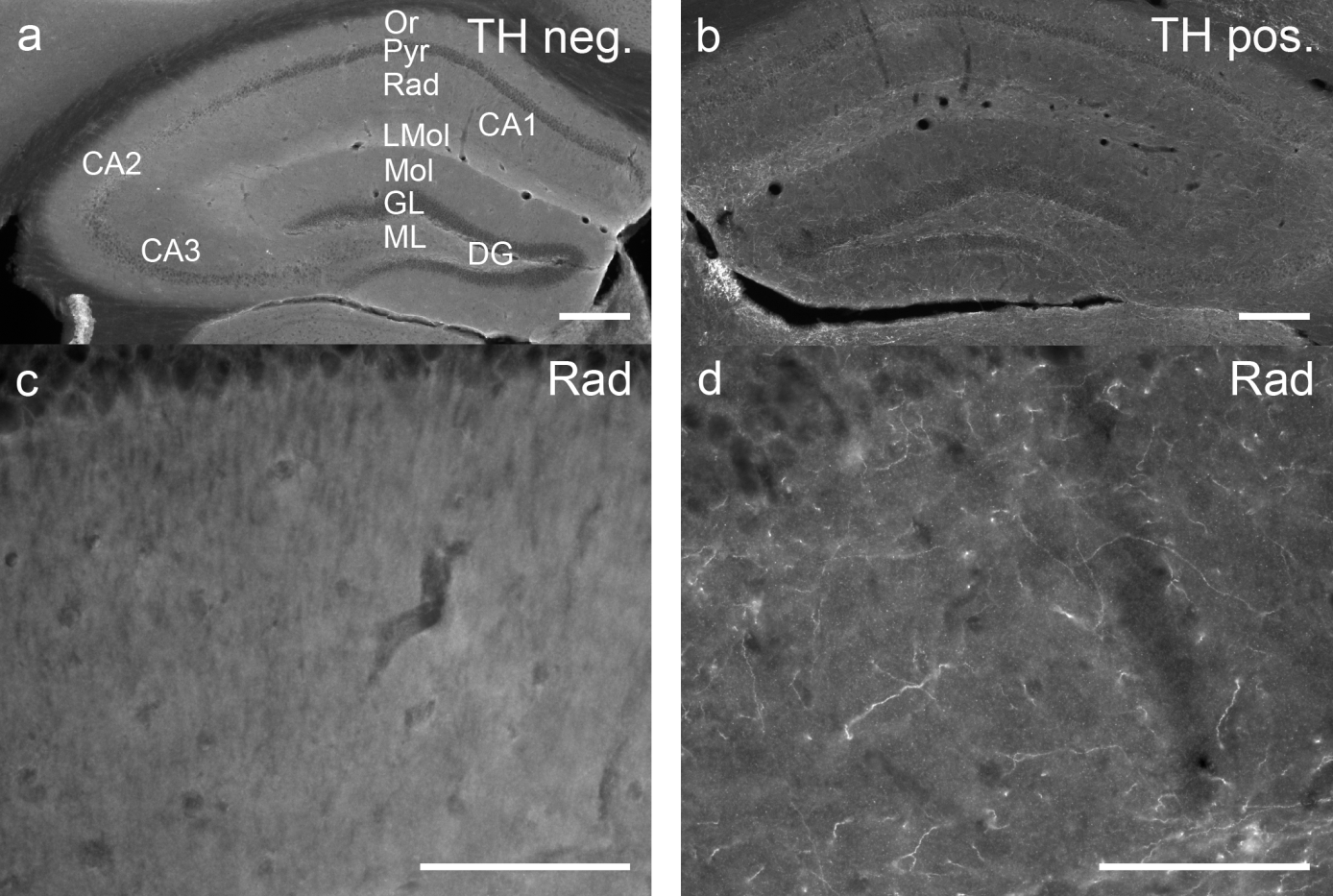
**

**Fig. S2.** Immunofluorescence staining with anti-tyrosine hydroxylase (TH) primary antibody (dilution 1:1000; ab112; Abcam, Cambridge, UK), showing TH-IR fibers in the hippocampus of a *GAD67^+/+^* mouse. The images on the top (**a**, **b**) give an overview of the hippocampal formation and their subregions, **c** and **d** illustrate the CA1 stratum radiatum (Rad). Slides **a** and **c** are negative controls (TH neg., omission of anti-TH primary antibody). Slides **b** and **d** show TH-IR staining (TH pos.). Scale bar: 100 µm. The specificity of the primary antibody for immunofluorescence is shown in Nullmeier et al. (2014), Spilker et al. (2016), Daigle et al. (2018) and Jeong et al. (2018).


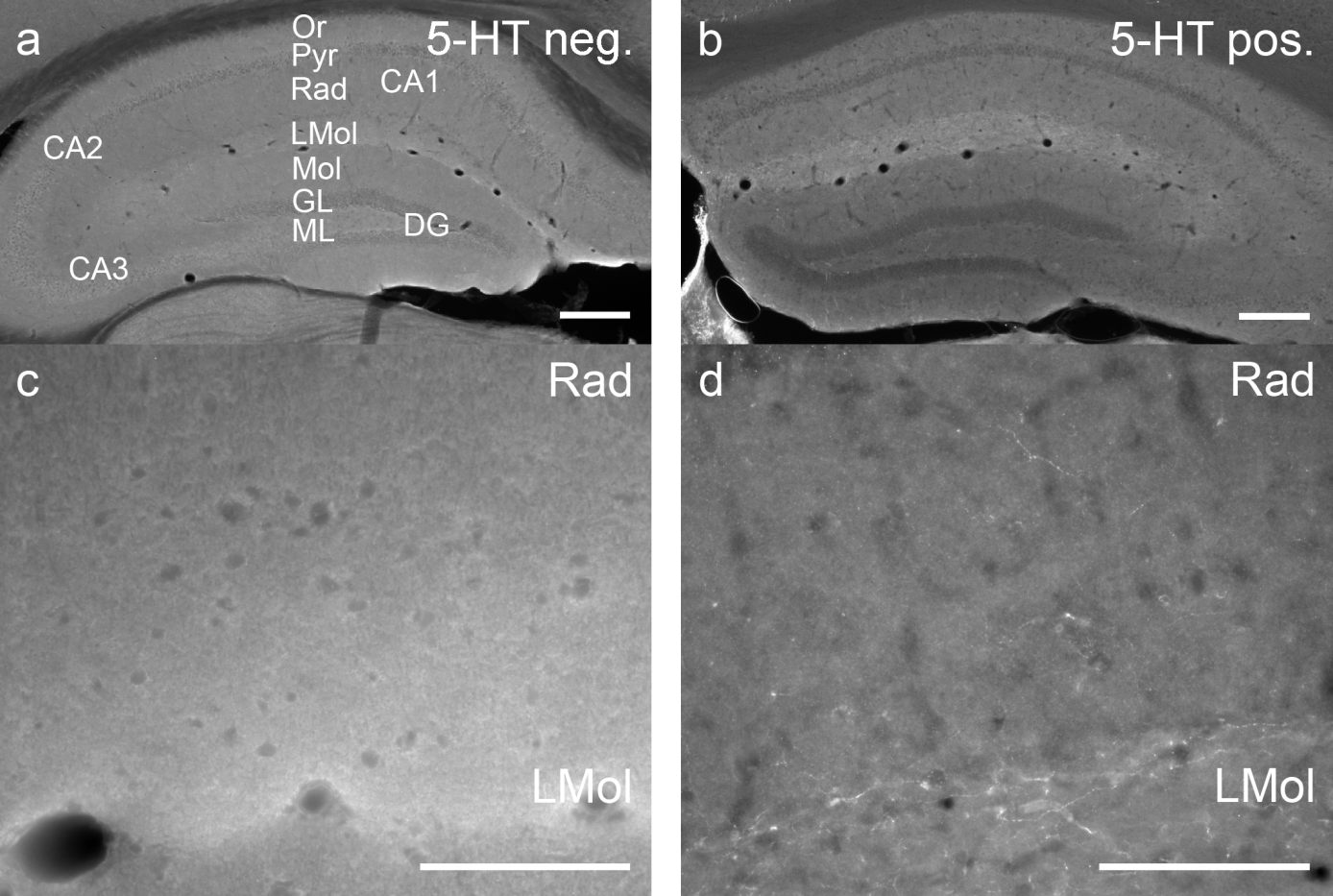


**Fig. S3.** Immunofluorescence staining with anti-serotonin (5-HT) primary antibody (dilution: 1:15000; catalog number: 20080; ImmunoStar, Hudson, WI, USA), showing 5-HT-IR fibers in the hippocampus of a *GAD67^+/+^* mouse. The images on the top (**a** and **b**) give an overview of the hippocampal formation and their subregions, **c** and **d** illustrate the CA1 stratum radiatum (Rad) and lacunosum moleculare (LMol). Slides **a** and **c** are negative controls (5-HT neg., omission of anti-5-HT primary antibody). Slides **b** and **d** show 5-HT-IR staining (5-HT pos.). Scale bar: 100 µm. The specificity of the primary antibody for immunofluorescence is shown in Kala et al. (2009), Nullmeier et al. (2014), Jeong et al. (2015) and Athilingam et al. (2017).


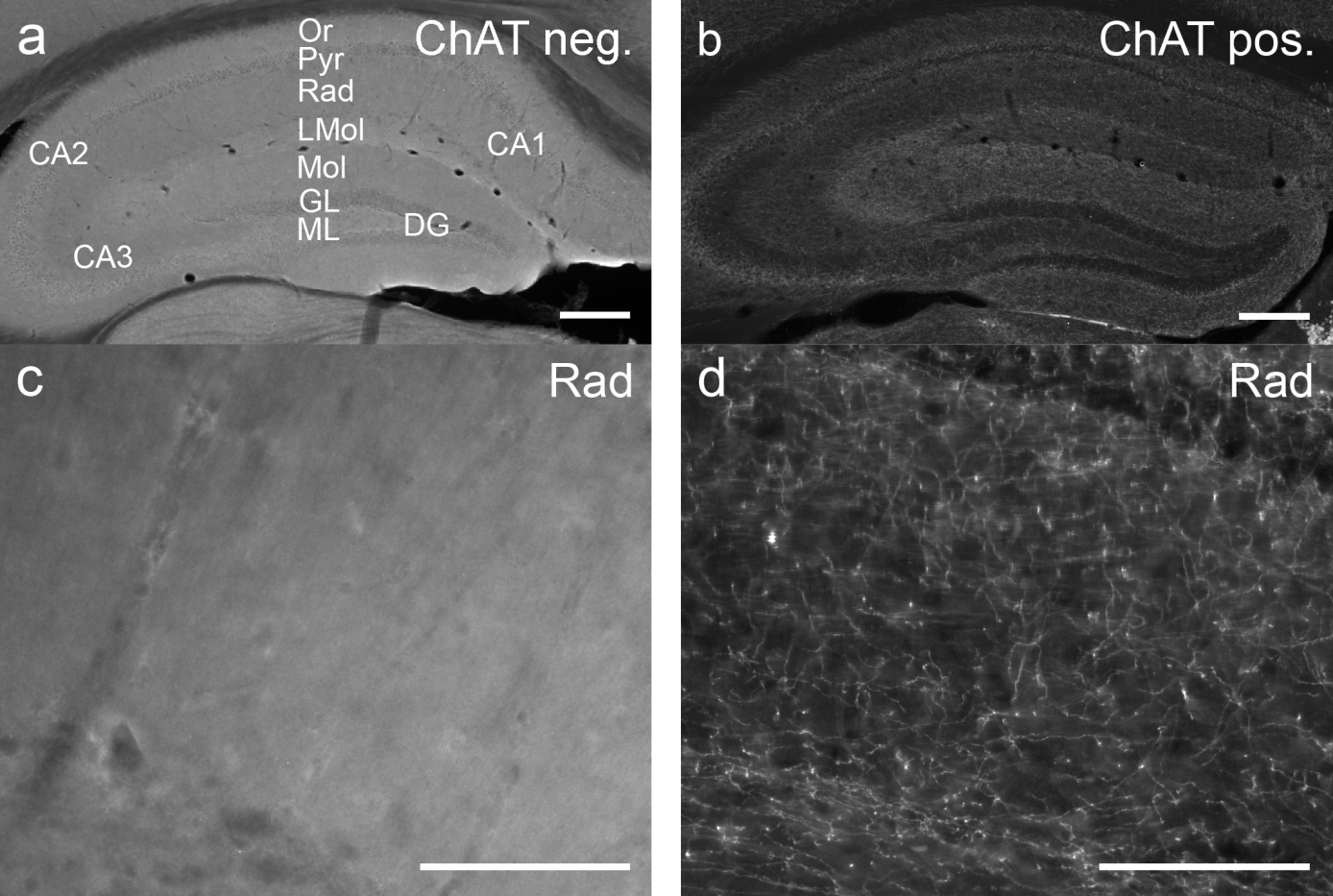


**Fig. S4.** Immunofluorescence staining with anti-choline acetyltransferase (ChAT) primary antibody (dilution: 1:100; Cat. #AB144P, Millipore, Billerica, MA, USA), showing ChAT-IR fibers in the hippocampus of a *GAD67^+/+^* mouse. The images on the top (**a** and **b**) give an overview of the hippocampal formation and their subregions, **c** and **d** illustrate the CA1 stratum radiatum (Rad). Slides **a** and **c** are negative controls (ChAT neg., omission of anti-ChAT primary antibody). Slides **b** and **d** show ChAT-IR staining (ChAT pos.). Scale bar: 100 µm. The specificity of the primary antibody for immunofluorescence is shown in Paez-Gonzalez et al. (2014) and Daigle et al. (2018).


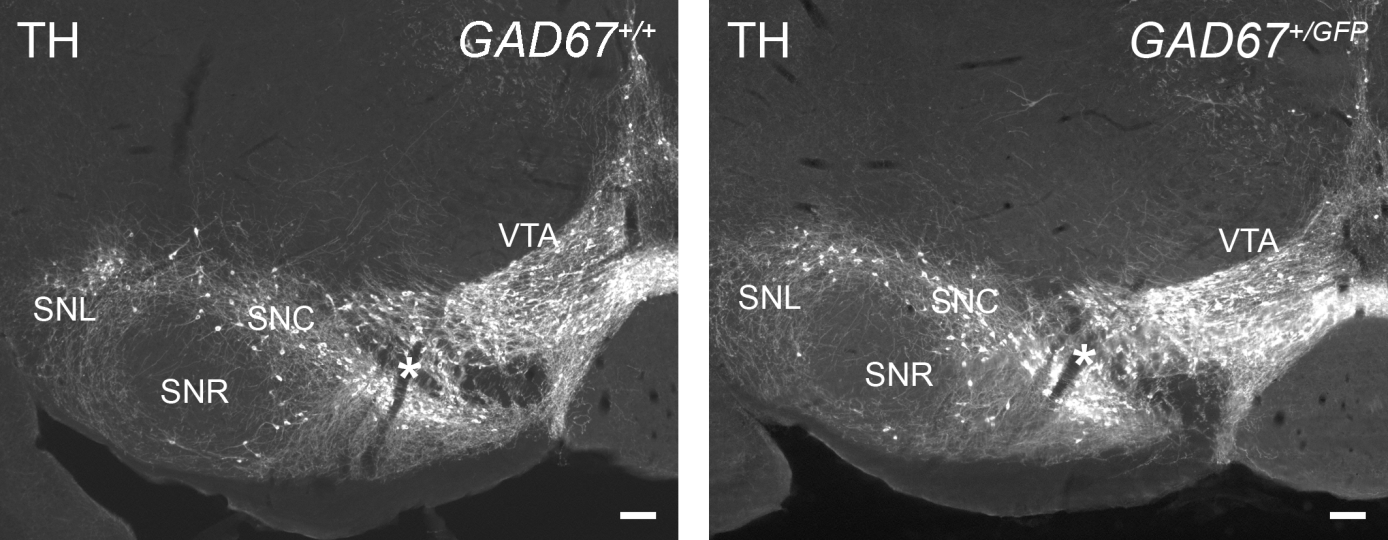


**Fig. S5.** Microphotographs of coronal sections (Bregma -3.28mm) illustrating the location of tyrosine hydroxylase (TH)-IR neurons in substantia nigra (SN) and ventral tegmental area (VTA) of *GAD67^+/+^* and *GAD67^+/GFP^* mice. The images are taken at the height of oculomotor nerve (asterisk). Scale bar: 100 µm. Abbreviations: SNC, substantia nigra pars compacta; SNL, substantia nigra pars lateralis; SNR, substantia nigra pars reticulata.


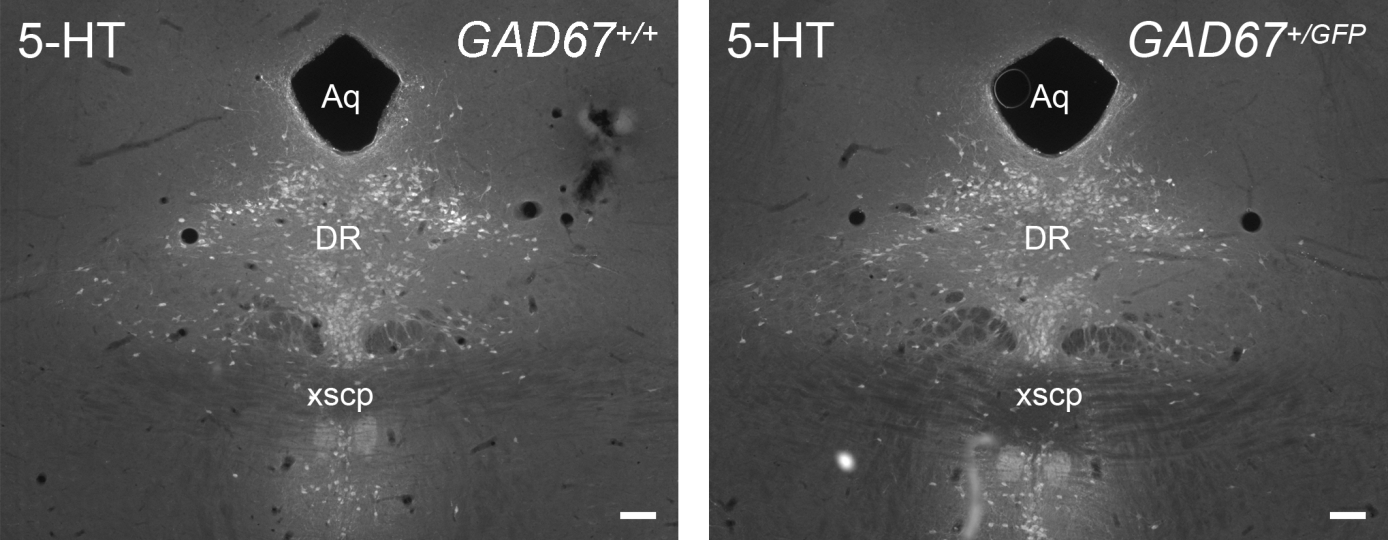


**Fig. S6.** Microphotographs of coronal sections (Bregma -4.48mm) illustrating the location of serotonergic (5-HT)-IR neurons in dorsal nucleus raphe (DR) of *GAD67^+/+^* and *GAD67^+/GFP^* mice. Scale bar: 100 µm. Abbreviations: Aq, cerebral aqueduct; xscp, decussation of superior cerebellar peduncle.


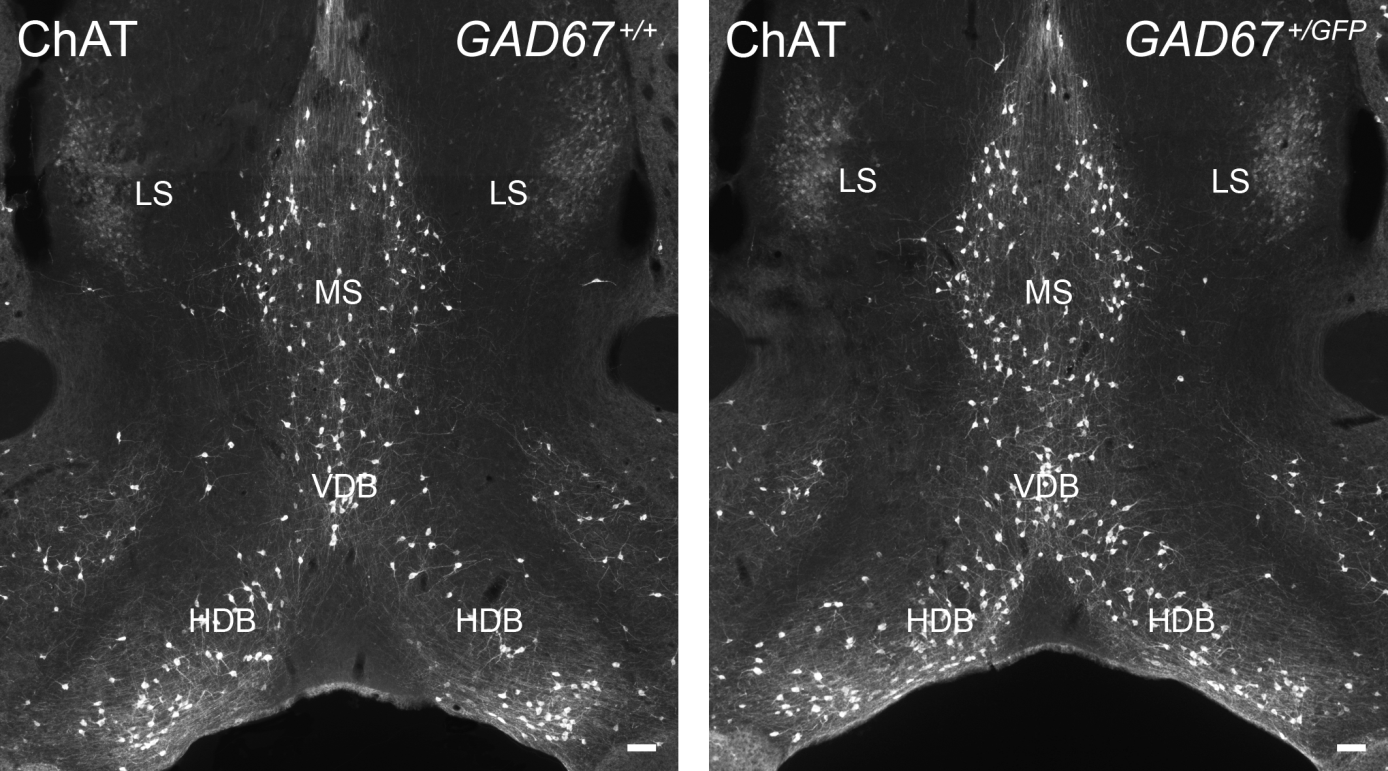


**Fig. S7.** Microphotographs of coronal sections (Bregma 0.74mm) illustrating the location of choline acetyltransferase (ChAT)-IR neurons in the septal region of *GAD67^+/+^* and *GAD67^+/GFP^* mice. Scale bar: 100 µm. Abbreviations: HDB, horizontal limb of the diagonal band of Broca; LS, lateral septal nucleus; MS, medial septal nucleus; VDB, vertical limb of the diagonal band of Broca.

# Supplementary material and methods

## Western blot analysis

Western immunoblotting was performed as previously described (Wiegreffe et al. 2015). In brief, prefrontal cortices and hippocampi of both hemispheres from 14-15 week old, male, group housed GAD67^+/+^ (n=4) and GAD67^+/GFP^ mice (n=4) were dissected on ice and lysed in RIPA lysis buffer (50mM Tris-HCL pH 7.5, 150mM NaCl, 0.5% deoxycholate, 1% NP-40, 0.1% SDS) supplemented with protease inhibitors (cOmplete™Mini, EDTA-free Protease Inhibitor, Roche). Lysates were centrifuged at 13000 rpm for 25 minutes at 4°C and supernatant was collected for Western blotting. Protein concentrations were determined using the Bradford method (Bradford 1976). Proteins (10µg per well) were separated by SDS-Page (10% acrylamide gel under reducing conditions) and transferred to PVDF membranes (Imobilion-P, Merck Millipore). Membranes were blocked (5% BSA in TBS+0.1% Tween for 1 hour at room temperature) and probed with mouse anti-GAD67 (MAB5406; 1:5000, Millipore) and mouse anti-β-actin (A5441, 1:4000, Sigma-Aldrich) primary antibodies overnight at 4°C. After washing (TBS+0.1% Tween for 3 x 5 minutes) membranes were incubated with horseradish peroxidase-conjugated secondary antibodies (Catalog Number: 715-035-151, 1:10000, Jackson ImmunoResearch) and ECL Plus western blotting detection reagents in accordance with the manufacturer’s instructions (ThermoScientific). Images were acquired with ChemiDoc imaging system (Bio-Rad) and quantification of the western blot band intensities was performed using Fijii software (http://fiji.sc). The GAD67 signal was normalized against β-actin level to control for variations in protein loading. Statistically analysis of GAD67 Western blots was carried out using unpaired *t* tests, respectively. Data are presented as means ± SEM. Alpha level was set at 0.05 for all main and interaction effects. The software package SPSS (IBM SPSS Statistics for Windows, Version 21.0. Armonk, NY: IBM Corp) was used for statistical analysis.

# Supplementary results

## Reduced GAD67 protein expression in *GAD67^+/GFP^* mice

Densitometric western blot analysis of GAD67 bands (Fig. S1a) normalized against housekeeping protein β-actin revealed, that (Fig. S1b) GAD67 protein is decreased by 33% in prefrontal cortex (*t =* 4.88, *df =* 6, *p <* 0.01) and by 32% in hippocampus (*t =* 3.77, *df =* 6, *p <* 0.01) of *GAD67^+/GFP^*, compared to *GAD67^+/+^* mice.

# Supplementary references

Athilingam JC, Ben-Shalom R, Keeshen CM, Sohal VS, Bender KJ (2017) Serotonin enhances excitability and gamma frequency temporal integration in mouse prefrontal fast-spiking interneurons. eLife 6. https://doi.org/10.7554/eLife.31991

Bradford MM (1976) A rapid and sensitive method for the quantitation of microgram quantities of protein utilizing the principle of protein-dye binding. Anal Biochem 72:248-254. https://doi.org/10.1006/abio.1976.9999

Daigle TL, Madisen L, Hage TA, Valley MT, Knoblich U, Larsen RS, Takeno MM, Huang L, Gu H, Larsen R, Mills M, Bosma-Moody A, Siverts LA, Walker M, Graybuck LT, Yao Z, Fong O, Nguyen TN, Garren E, Lenz GH, Chavarha M, Pendergraft J, Harrington J, Hirokawa KE, Harris JA, Nicovich PR, McGraw MJ, Ollerenshaw DR, Smith KA, Baker CA, Ting JT, Sunkin SM, Lecoq J, Lin MZ, Boyden ES, Murphy GJ, da Costa NM, Waters J, Li L, Tasic B, Zeng H (2018) A Suite of Transgenic Driver and Reporter Mouse Lines with Enhanced Brain-Cell-Type Targeting and Functionality. Cell 174 (2):465-480.e422. https://doi.org/10.1016/j.cell.2018.06.035

Jeong GR, Jang E-H, Bae JR, Jun S, Kang HC, Park C-H, Shin J-H, Yamamoto Y, Tanaka-Yamamoto K, Dawson VL, Dawson TM, Hur E-M, Lee BD (2018) Dysregulated phosphorylation of Rab GTPases by LRRK2 induces neurodegeneration. Molecular Neurodegeneration 13 (1):8. https://doi.org/10.1186/s13024-018-0240-1

Jeong JH, Lee DK, Blouet C, Ruiz HH, Buettner C, Chua S, Jr., Schwartz GJ, Jo YH (2015) Cholinergic neurons in the dorsomedial hypothalamus regulate mouse brown adipose tissue metabolism. Molecular metabolism 4 (6):483-492. https://doi.org/10.1016/j.molmet.2015.03.006

Kala K, Haugas M, Lillevali K, Guimera J, Wurst W, Salminen M, Partanen J (2009) Gata2 is a tissue-specific post-mitotic selector gene for midbrain GABAergic neurons. Development 136 (2):253-262. https://doi.org/10.1242/dev.029900

Nullmeier S, Panther P, Frotscher M, Zhao S, Schwegler H (2014) Alterations in the hippocampal and striatal catecholaminergic fiber densities of heterozygous reeler mice. Neuroscience 275:404-419. https://doi.org/10.1016/j.neuroscience.2014.06.027

Paez-Gonzalez P, Asrican B, Rodriguez E, Kuo CT (2014) Identification of distinct ChAT+ neurons and activity-dependent control of postnatal SVZ neurogenesis. Nature Neuroscience 17:934. https://doi.org/10.1038/nn.3734

https://www.nature.com/articles/nn.3734#supplementary-information

Spilker C, Nullmeier S, Grochowska KM, Schumacher A, Butnaru I, Macharadze T, Gomes GM, Yuanxiang P, Bayraktar G, Rodenstein C, Geiseler C, Kolodziej A, Lopez-Rojas J, Montag D, Angenstein F, Bar J, D'Hanis W, Roskoden T, Mikhaylova M, Budinger E, Ohl FW, Stork O, Zenclussen AC, Karpova A, Schwegler H, Kreutz MR (2016) A Jacob/Nsmf Gene Knockout Results in Hippocampal Dysplasia and Impaired BDNF Signaling in Dendritogenesis. PLoS Genet 12 (3):e1005907. https://doi.org/10.1371/journal.pgen.1005907

Wiegreffe C, Simon R, Peschkes K, Kling C, Strehle M, Cheng J, Srivatsa S, Liu P, Jenkins NA, Copeland NG, Tarabykin V, Britsch S (2015) Bcl11a (Ctip1) Controls Migration of Cortical Projection Neurons through Regulation of Sema3c. Neuron 87 (2):311-325. https://doi.org/10.1016/j.neuron.2015.06.023
